# Suppression of PTRF Alleviates Post-Infectious Irritable Bowel Syndrome via Downregulation of the TLR4 Pathway in Rats

**DOI:** 10.3389/fphar.2021.724410

**Published:** 2021-10-07

**Authors:** Hui-hui Zhou, Ye-ming Zhang, Sheng-peng Zhang, Qi-xiang Xu, Ya-qing Tian, Ping Li, Di Cao, Yong-qiu Zheng

**Affiliations:** Provincial Engineering Laboratory for Screening and Re-evaluation of Active Compounds of Herbal Medicines in Southern Anhui, Teaching and Research Section of Traditional Chinese Medicine, School of Pharmacy, Wannan Medical College, Wuhu, China

**Keywords:** polymerase I and transcript release factor, post-infectious irritable bowel syndrome, TLR4 signaling, ERK, JNK, p38, iNOS

## Abstract

**Background:** Accumulating evidence suggests that the polymerase I and transcript release factor (PTRF), a key component of the caveolae structure on the plasma membrane, plays a pivotal role in suppressing the progression of colorectal cancers. However, the role of PTRF in the development of functional gastrointestinal (GI) disorders remains unclear. Post-infectious irritable bowel syndrome (PI-IBS) is a common functional GI disorder that occurs after an acute GI infection. Here, we focused on the role of PTRF in the occurrence of PI-IBS and investigated the underlying mechanisms.

**Methods:** Lipopolysaccharide (LPS) (5 μg/ml) was used to induce inflammatory injury in human primary colonic epithelial cells (HCoEpiCs). Furthermore, a rat model of PI-IBS was used to study the role of PTRF. Intestinal sensitivity was assessed based on the fecal water content. A two-bottle sucrose intake test was used to evaluate behavioral changes. Furthermore, shRNA-mediated knockdown of PTRF was performed both *in vitro* and *in vivo*. We detected the expression of PTRF in colonic mucosal tissues through immunohistochemistry (IHC), western blotting (WB), and immunofluorescence (IF) analysis. Luciferase activity was quantified using a luciferase assay. Co-localization of PTRF and Toll-like receptor 4 (TLR4) was detected using IF analysis. The activation of the signaling pathways downstream of TLR4, including the iNOs, p38, extracellular signal-regulated kinase (ERK), and c-Jun N-terminal kinase (JNK) pathways, was detected via WB. The levels of NO, IL-1β, IL-6, and TNF-α were measured using enzyme-linked immunosorbent assays.

**Results:** LPS significantly induced PTRF expression and signaling downstream of TLR4, including p38, ERK, and JNK pathways, in HCoEpiCs. Moreover, shRNA-mediated knockdown of PTRF in HCoEpiCs significantly decreased the phosphorylation of JNK, ERK, and p38 and iNOS expression. In PI-IBS rats, the lack of PTRF not only reduced fecal water content and suppressed depressive behavior but also increased the body weight. Furthermore, we found a strong co-localization pattern for PTRF and TLR4. Consistently, the lack of PTRF impaired TLR4 signaling, as shown by the decreased levels of p-JNK, p-ERK, and p-p38, which are upstream factors involved in iNOS expression.

**Conclusion:** PTRF promoted PI-IBS and stimulated TLR4 signaling both *in vitro* and *in vivo*. The results of this study not only enlighten the pathogenesis of PI-IBS but also help us understand the biological activity of PTRF and provide an important basis for the clinical treatment of PI-IBS by targeting PTRF.

## Introduction

More than 40% of the people worldwide suffer from functional gastrointestinal (GI) disorders, which seriously threaten the quality of life of these patients and result in high social costs (Fikree and Byrne, 2021; Sperber et al., 2021). Irritable bowel syndrome (IBS) is a chronic, recurring, and remitting functional disorder of the GI tract associated with multiple comorbidities such as dyspepsia, interstitial cystitis, fibromyalgia, insomnia, chronic fatigue, headache/migraine, and psychiatric disturbances (Chang, 2014). In post-infectious IBS (PI-IBS), a subset of IBS, gastroenterologists often encounter situations where the pathologies of functional and organic disorders overlap, which is described as the “blurring of boundaries.” An initiating GI infection is required for the development of PI-IBS. The key associating features of PI-IBS include pain, diarrheal symptoms, and microscopic inflammation, which are associated with psychological distress (Grover et al., 2009). Thus, as a common GI disorder, PI-IBS is characterized by gut flora, immune dysregulation, and the effect of stress (Gong et al., 2014).

Although there are several drugs for PI-IBS, effective and approved treatments for one or more of the symptoms of PI-IBS are needed. An improved understanding of the pathophysiological mechanisms such as the role of the impaired bile acid metabolism, neurohormonal regulation, immune dysfunction, the role of the epithelial barrier, and psychological distress of the gut has led to advancements in the treatment of IBS (Saha, 2014).

The therapeutic effects of medicines against PI-IBS are hard to evaluate due to the lack of preclinical animal models (Bensoussan et al., 1998; Brandt et al., 2009). In this study, a rat model of PI-IBS with associated symptoms of visceral hypersensitivity, high fecal water content, anxiety, depression, and slight colitis was established through complex stimulation with early postnatal sibling deprivation (EPSD), intrarectal administration of trinitro-benzene-sulfonic acid (TNBS), and chronic unpredictable mild stress (CUMS) (Wang et al., 2015b).

Furthermore, the molecular pathophysiological mechanisms of PI-IBS remain unclear (Liu et al., 2014; Liu et al., 2020). Accumulating evidence shows that persistent inflammation is a common phenomenon in a variety of IBS subtypes as the gut microbiota precipitates the onset of colitis. The levels of inflammatory cytokines TNF-α, IL-1β, and IL-12 were found to be elevated in the intestinal tissue (Zhang et al., 2019; Camilleri, 2021). However, no specific therapeutic strategy targeting these molecules has been identified to date.

Toll-like receptors (TLRs) are vital for maintaining tissue homeostasis and immune tolerance (Mokhtari et al., 2021). TLR2 heterodimeric complexes have been reported to play a role in the modulation of the immune system and the pathogenesis of intestinal inflammation (Al-Sadi et al., 2021; Dejban et al., 2021). It is well acknowledged that among the TLRs, TLR4 is the receptor for lipopolysaccharide (LPS) (Newton and Dixit, 2012). It has been reported that MYD88-, TLR2-, TLR4-, TLR5-, and TLR9-deficient mice are more susceptible to chemically induced colitis owing to defects in intestinal epithelial permeability, reduced proliferation, and increased apoptosis (Burgueño et al., 2020). Furthermore, suppression of TLR4 signaling resulted in the development of necrotizing enterocolitis (Sodhi et al., 2021).

Accumulating evidence has shown that some caveolae-associated proteins, such as cavin-1, cavin-2, cavin-3, and cavin-4, were involved in regulating cell growth, endocytosis, mitochondrial functions, migration, and senescence (Hill et al., 2008; Bai et al., 2020). Cavin-1, also known as the polymerase 1 and transcript release factor (PTRF), is a conserved structural protein that regulates caveolae functions (Hill et al., 2008). Previous research has shown that PTRF participates in some key pathways during the progression of intestinal diseases. Wang et al. discovered that PTRF inhibits the metabolic processes of colorectal cancer cells, including proliferation, migration, and invasion (Wang et al., 2017a). However, it remains unknown whether PTRF has any influence on the development of the PI-IBS disorder. Moreover, the lack of PTRF impaired the formation of the TLR4/Myd88 complex, while LPS strengthened the co-localization and the interaction between PTRF and TLR4 in lipid rafts (Zheng et al., 2013). The detailed role of TLR4 signaling in the PI-IBS disorder and its connection with PTRF remain to be identified.

In this study, inflammation was induced in HCoEpiCs by LPS to characterize the expression of PTRF and investigate its role in alleviating inflammation. In addition, a PI-IBS rat model was simultaneously established to identify the role of PTRF in the occurrence of PI-IBS and to analyze the underlying mechanisms.

## Materials and Methods

### Cell Cultures and Treatment

Human primary colonic epithelial cells (HCoEpiCs) were obtained from ScienCell and cultured in the colonic epithelial cell medium supplemented with 100 μg/ml penicillin, 100 μg/ml streptomycin, and 10% fetal bovine serum at 37°C in a humidified atmosphere with 5% (v/v) CO_2_. The cells were treated with LPS (5 μg/ml) for 6 h after adherence to the wall.

### Establishment of Stably Transfected Cells

For knockdown studies, the target sequence in the PTRF cDNA was selected, and the appropriate shRNA oligo was cloned into the GV594-U6-MCS-CAG-firefly_Luciferase vector. The sh PTRF sense (S) oligonucleotide sequence 5′-GCC​AGA​TAA​AGA​AAC​TGG​AGG​TCA​A and the sh PTRF antisense (As) oligonucleotide sequence 5′-TTG​ACC​TGG​AGT​TTC​TTT​ATC​TGG​C were annealed and cloned into a pGC/U6 plasmid. One unrelated sequence (S: 5′-GCC​AAT​AAA​GAG​TCA​GGA​GTG​ACA​A and As: 5′-TTG​TCA​CTC​CTG​ACT​CTT​TAT​TGG​C) was used as a control (GENECHEM Biotech, Shanghai, CN). Cells were transfected with a specific plasmid or control vector using Lipofectamine 2000 according to the manufacturer’s instructions (Invitrogen, United States) and retained in a medium containing 10% fetal bovine serum.

### Animals

Adult pregnant Sprague–Dawley rats weighing 190–210 g were purchased from Zhejiang Weitong Lihua Laboratory Animal Technology Co., Ltd. (animal license number SCXK; Zhejiang; 2019-0001). All rats were supplied by experimental protocols in accordance with the National Institutes of Health regulations program, and the study was approved by the Institutional Animal Care and Use Committee of Wannan Medical College. Throughout the acclimatization and study periods, all animals had access to food and water *ad libitum* and were maintained on a 12 h light/dark cycle (at 21 ± 2°C with a relative humidity of 45 ± 10%), except during the CUMS and sucrose intake test periods. The rats were housed under specific pathogen-free conditions.

### PI-IBS Rat Model Establishment

Because PI-IBS is an inflammatory immune disease, only newborn male rats were used. According to reported procedures (Wang et al., 2015b; Ma et al., 2017), a rat model of PI-IBS was established using a multi-stimulation paradigm including EPSD and intrarectal administration of TNBS and CUMS.

**FIGURE 1:**
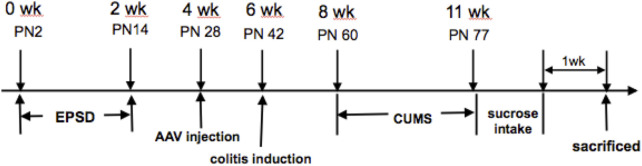
Experimental protocol.

Briefly, litters were removed from their maternity cages to the adjacent cages at 9:00–12:00 am from postnatal day 2 (PN2) to postnatal day 14. Two weeks after adeno-associated virus (AAV) injection (on PN28), colitis was induced in rats after pentobarbital anesthesia based on a previously published method, which involved the intrarectal administration of 0.8 ml of TNBS solution (20 mg per rat) in 50% ethanol on postnatal day 42 (Qin et al., 2012). The control rats were administered with 0.8 ml of 50% ethanol as a vehicle. All solutions were delivered via a soft catheter, introduced 8 cm above the anus. After recovery from TNBS modeling for 2 weeks, the protocol was performed as follows: 1) water fasting for 24 h, 2) fasting for 24 h, 3) reverse day/night cycle (dark treatment from 7:00 to 19:00 and lighting from 19:00 to 7:00 the next day), 4) cold stress (rats were put into a transparent barrel containing ice water at 4°C at a depth of 15 cm) for 5 min, 5) heat stress (rats were put into a thermostat at 45°C) for 5 min, 6) pain induction (rats were put into an observation cage, and their tails were clipped at 1 cm from the distal tip, with appropriate strength to make the rats scream), and 7) horizontal oscillation [rats were placed into a horizontal oscillator at a high speed (110/min)] for 15 min. Each type of stress was performed daily for 21 consecutive days, as shown in Figure 1 (Mineur et al., 2006; Isingrini et al., 2010).

### AAV9-PTRF-KD Construction and Tail Vein Injection

For the PTRF knockdown studies, rats were randomly divided into three groups (*n* = 6 per group): normal, PI-IBS/AAV9-PTRF-KD, and PI-IBS/AAV9-NC. PTRF knockdown and control recombinant AAV9-luciferase vectors were constructed (GENECHEM Biotech). The administration procedures were performed according to previous studies (Fang et al., 2019). Briefly, 5 × 10^10^ physical particles of AAV in 200 μl of PBS were injected into the tail veins of rats in the PI-IBS/AAV9-PTRF-KD and PI-IBS/AAV-NC groups.

### Luciferase Reporting Assay

Luciferase activity in plasma was quantified using a luciferase assay system (Promega, Madison, WI, United States) according to the manufacturer’s protocol. One week after the sucrose intake test, the colon was removed for a PTRF expression evaluation by western blotting (WB) and immunohistochemistry (IHC). The results were reported as luciferase activity/total protein.

### Sucrose Intake Test

To evaluate the effect of PTRF on depression, a sucrose intake (1% sucrose solution) test was performed as described in a previous study (Ribeiro-Carvalho et al., 2011). Rats were singly housed and supplied with one bottle filled with tap water and another with 2% sucrose solution for 24 h. To balance side preference, locations of the two bottles were switched after 12 h. Following 24 h of fasting and water abstinence, the experimental process was initiated immediately after the training completion. Rats were given 1 h access to sucrose solution and water, and the consumption of each bottle was recorded. The percentage of sucrose consumption was calculated as (sucrose intake/total intake) × 100.

### Histology

One week after the sucrose intake test, rat colons were removed and fixed in paraformaldehyde. The colons were then embedded in paraffin and cut into 5 μm-thick sections. For hematoxylin and eosin (H&E) staining, the slices were developed using 3,3′-diaminobenzidine (DAB) and counterstained with hematoxylin.

### Immunohistochemical Staining

The colon samples were fixed in 4% formalin and embedded in paraffin. Tissue slices of a 5 μm thickness were prepared for IHC staining. The PTRF antibody (Thermo Fisher Scientific) at a dilution of 1:200 was used as the primary antibody. For IHC assessment, the entire tissue section was scanned and scored by two independent pathologists.

### Immunofluorescence

Cells were cultured in confocal dishes and washed with PBS three times, followed by fixation in 4% paraformaldehyde for 30 min, permeabilization with 0.5% Triton X-100, and blocking with goat serum. Then, the cells were subjected to staining with the anti-PTRF antibody (Thermo Invitrogen) at a dilution of 1:50, followed by the CY3-conjugated goat anti-rabbit secondary antibody (Abcam) at a dilution of 1:500 prior to imaging. For paraffin slices obtained from colon tissue samples, the PTRF antibody (1:50, Thermo Invitrogen) and TLR4 antibody (1:50, Thermo Invitrogen) were used as primary antibodies, followed by the FITC-conjugated goat anti-mouse secondary antibody (1:500, Abcam) or the CY3-conjugated goat anti-rabbit secondary antibody (1:500, Abcam) for imaging. The cells and slides were then counterstained with 4′,6-diamidino-2-phenylindole (DAPI) as a nuclear indicator and visualized with a confocal laser-scanning microscope (LSM 880 Carl Zeiss).

### ELISA

Colon homogenates were centrifuged at 10,000 *xg* for 20 min, and protein concentrations in the supernatants of the homogenates were determined using a bicinchoninic acid assay kit. Tissue homogenates containing TNF-α, IL-6, IL-1, and NO were centrifuged at 1,000 × *g*, 4°C for 10 min, and the concentrations of these cytokines in the supernatants were measured using enzyme-linked immunosorbent assay (ELISA) kits, according to the manufacturer’s directions (R&D systems). The concentrations of NO were measured using a total nitric oxide assay kit (Beyotime Biotechnology). The sample and standard dilutions were prepared using the experimental media, and the results were expressed as the mean ± standard deviation (SD).

### Western Blot Analysis

Proteins were extracted from tissue samples or cell lysates using RIPA buffer [150 mM NaCl, 50 mM Tris-Cl, 1 mM EGTA, 1% (v/v) Triton X-100, 0.1% (w/v) sodium dodecyl sulfate (SDS), and 1% (w/v) sodium deoxycholate, pH 8.0]. To test the expression profile of PTRF at different stages of PI-IBS, 12 rats were sacrificed at P2, P15 (after EPSD treatment), P50 (after EPSD and TNBS treatments), and P78 (after EPSD, TNBS, and CUMS treatments), and colon homogenates were prepared. Protein concentrations were determined using a protein assay solution (Bio-Rad). Equivalent amounts of proteins were denatured in the protein loading buffer, run on 10% SDS-PAGE gels, and subsequently transferred to polyvinylidene difluoride (PVDF) membranes (Millipore, Billerica, MA) by electroblotting. The PVDF membranes were blocked with 5% nonfat milk in Tris-buffered saline/Tween buffer for 1 h and incubated overnight at 4°C with antibodies against PTRF (1:2,000, Abcam), extracellular signal-regulated kinase (ERK) (1:2,000, Cell Signaling), p-ERK (1:1,000, Cell Signaling), c-Jun N-terminal kinase (JNK) (1:2,000, Cell Signaling), p-JNK (1:1,000, Cell Signaling), P38 (1:2,000, Cell Signaling), p-P38 (1:2,000, Cell Signaling), GAPDH (Cell Signaling, 1:2,000), and iNOS (1:1,000, Thermo Invitrogen). Signals were detected using an ECL detection reagent (Pierce, Inc.), following the manufacturer’s instructions.

### Data Quantification and Statistical Analysis

All data were presented as the mean ± SD. Statistical significance was analyzed using one-way analysis of variance (ANOVA), followed by Tukey’s test for multiple comparisons. A nonparametric test was used to compare band density values between the groups. Statistical significance was set at *p* < 0.05.

## Results

### PTRF was Required for LPS-Mediated iNOS Induction and NO Production in Colorectal Epithelial Cells

To investigate the potential role of PTRF in the regulation of PI-IBS, we studied several colorectal cell lines (Colo320, HT29, and CaCo_2_) and normal cells. Among these cell lines, the PTRF expression was the lowest in Colo320 cells and the highest in HCoEpiCs (Wang et al., 2017a). We then tested whether the differential expression of PTRF would lead to differences in the development of inflammation in HCoEpiCs.

**FIGURE 2:**
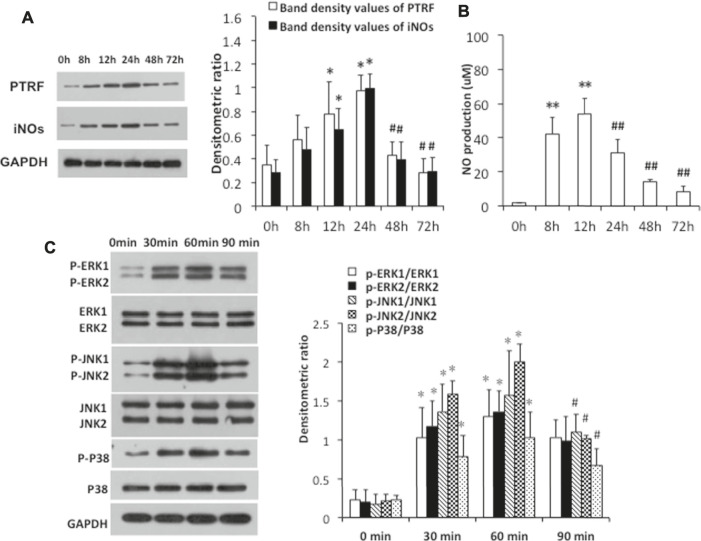
LPS-induced PTRF expression and iNOs pathway activation in HCoEpiCs. The cells were stimulated with LPS (5 μg/ml) for the indicated time intervals. **(A)** Cell lysates were blotted with anti-PTRF and anti-iNOs. The relative expressions were standardized to the endogenous control GAPDH. **Left:** Representative images of PTRF and iNOs expression; an antibody for GAPDH was used to show equal protein loading; right: bar graphs show quantitative evaluation (*n* = 3). Data are reported as the mean ± S.D., *p < 0.05 compared with 0 h, and ^#^ *p* < 0.05 compared with 24 h. **(B)** Nitric oxide (NO) production from supernatants of the cells was measured by ELISA. Data are reported as the mean ± S.D. *n* = 10, **p < 0.01 compared with 0 h, and ^##^ *p* < 0.01 compared with 24 h. **(C)** Cell lysates were blotted with anti-p38/phospho-p38, anti -ERK/p-ERK, and anti-JNK/p-JNK. The relative expressions were standardized to the endogenous control GAPDH. **Left:** representative images of p38/phospho-p38, ERK/p-ERK, and JNK/p-JNK blots; an antibody for GAPDH was used to show equal protein loading; right: bar graphs show quantitative evaluation (*n* = 3). Data are reported as the mean ± S.D., *p < 0.05 compared with 0 min, and ^#^ *p* < 0.05 compared with 60 min.

**FIGURE 3:**
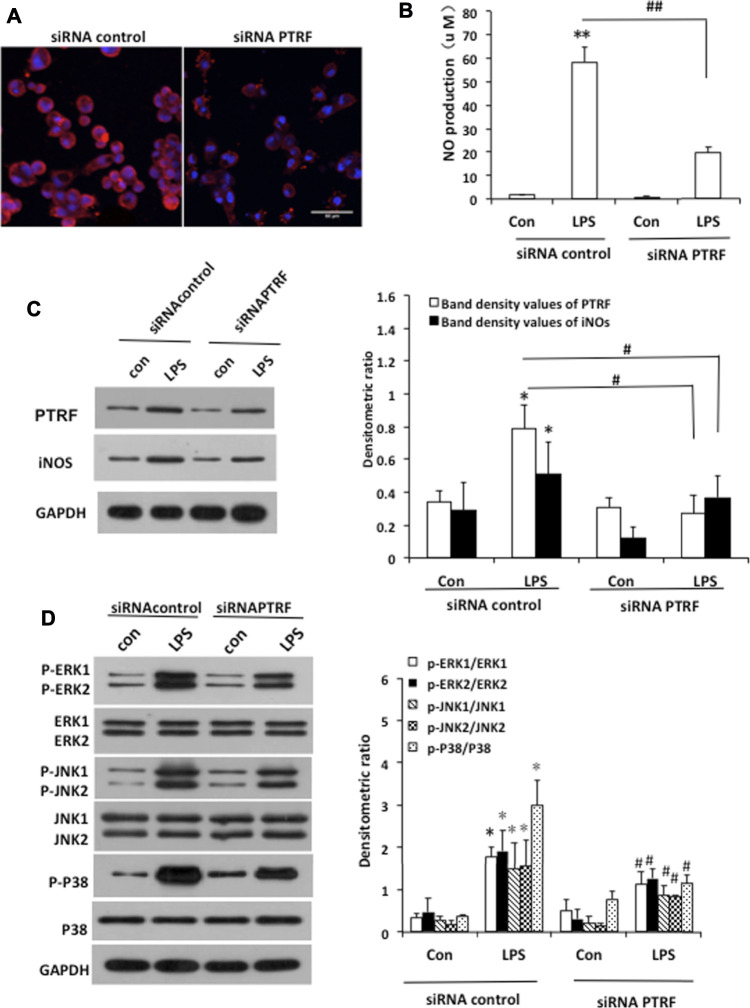
Deletion of PTRF inhibited LPS-induced iNOS pathway activation in HCoEpiCs. **(A)** Staining of PTRF (red) in HCoEpiCs transfected with the PTRF shRNA plasmid (siRNA PTRF) and negative control plasmid (siRNA control). Scale bar = 50 μm. **(B)** Levels of NO in the culture supernatant. *n* = 10, **p < 0.01 compared with the siRNA control, and ^##^ *p* < 0.01 compared with the LPS+siRNA control. **(C)** Western blot analysis of PTRF and iNOS in HCoEpiCs transfected with siRNA PTRF or the siRNA control. **Left:** representative images of PTRF and iNOs expression; an antibody for DAPDH was used to show equal protein loading; **right:** bar graphs show quantitative evaluation (*n* = 3). **(D)** Phosphorylation of ERK, JNK, and p38 in HCoEpiCs transfected with siRNA PTRF or siRNA control. **Left:** representative images of p38/phospho-p38, ERK/p-ERK, and JNK/p-JNK blots; an antibody for DAPDH was used to show equal protein loading; **right:** bar graphs show quantitative evaluation (*n* = 3). *p < 0.05 compared with the siRNA control, and ^#^ *p* < 0.05 compared with the LPS+siRNA control.

To further investigate the mechanisms by which PTRF regulates inflammation, we used LPS-induced HCoEpiCs as our cellular model *in vitro*. PTRF and iNOS expressions were significantly increased in HCoEpiCs, particularly within the first 24 h after LPS stimulation (Figure 2A). Consistent with these results, the level of NO was increased in the culture supernatant and peaked at 12 h (Figure 2B). Additional studies indicated that LPS induces the phosphorylation of mitogen-activated protein kinase (MAPK) signaling cascades, including ERK, p38, and JNK, which peaked at 90 min. Consequently, we deleted PTRF in HCoEpiCs using PTRF shRNA transfection. A PTRF deletion efficiency of more than 50% was achieved (Figures 3A,C). As shown in Figure 3C, siRNA-PTRF cells showed reduced iNOS expression, evidenced by WB analysis. Furthermore, NO, the product of iNOS, was significantly decreased in response to LPS in PTRF-knockdown cells (Figure 3B). Similarly, in HCoEpiCs, knockdown of PTRF significantly inhibited the phosphorylation of ERK, p38, and JNK as compared to control HCoEpiCs. These results suggested that PTRF indeed regulated LPS-induced inflammation in HCoEpiCs.

### AAV-Mediated PTRF Knockdown Resulted in Reduced TLR4/PTRF Interactions and Suppressed p38, ERK, and JNK Pathway Activation in PI-IBS rats

**FIGURE 4:**
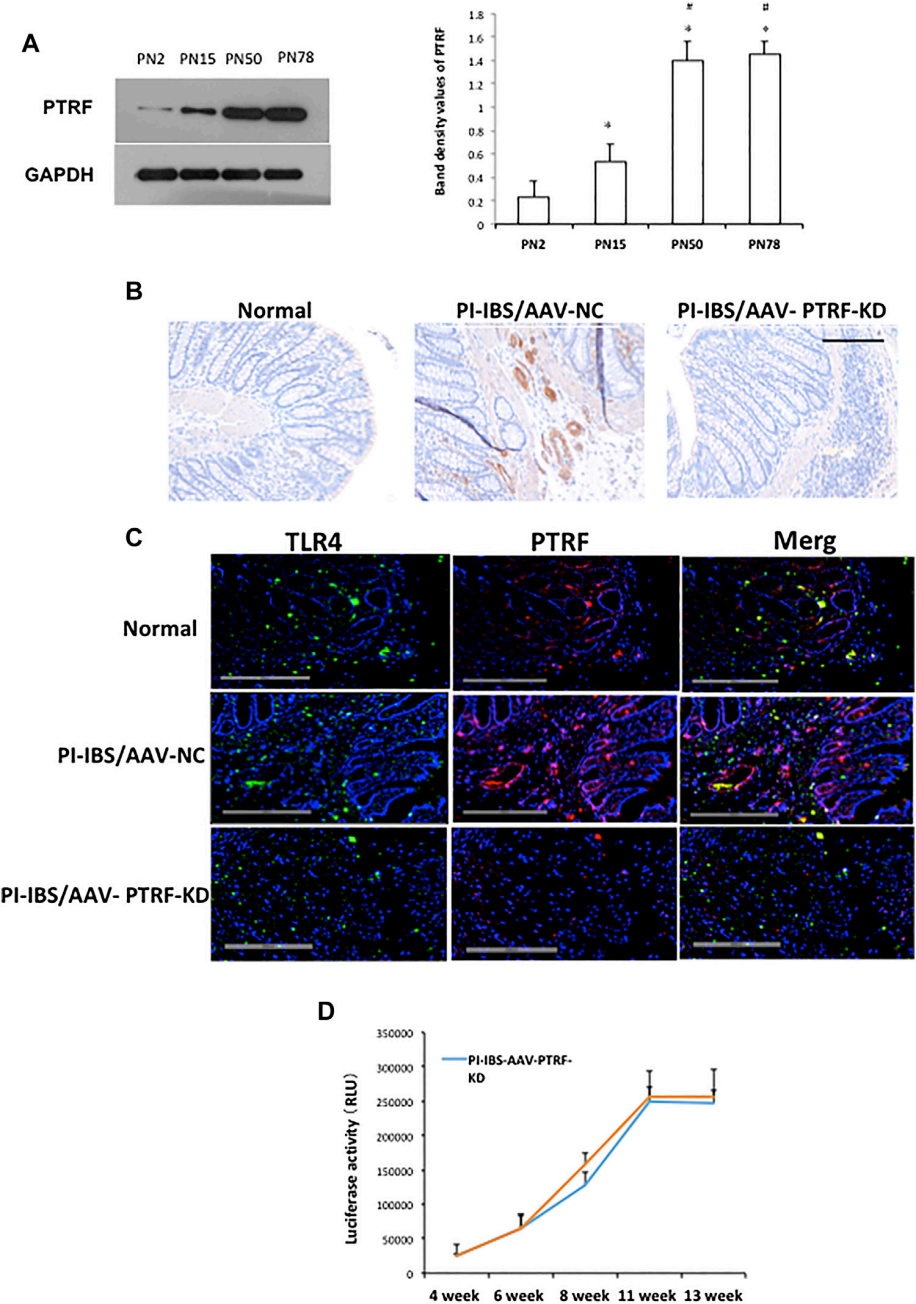
PTRF expression in rat colons following tail vein injection of the AAV9-PTRF-KD vector. **(A)** Homogenates of colons were blotted with anti-PTRF. The relative expressions were standardized to the endogenous control GAPDH. **Left:** representative image of PTRF expression; an antibody for DAPDH was used to show equal protein loading; **right:** bar graphs show quantitative evaluation (*n* = 3). Data are reported as the mean ± S.D., *p < 0.05 compared to PN2. ^#^ *p* < 0.05 compared to PN15. **(B)** Immunohistochemical staining of PTRF. **(C)** Merged confocal images represent overlays of PTRF (red), TLR4 (green), and nuclear staining by DAPI (blue). **(D)** Quantitative analysis of luciferase activity. *n* = 6, Mean ± SD. Scale bar = 200 μm in **(B)** and **(C)**.

**FIGURE 5:**
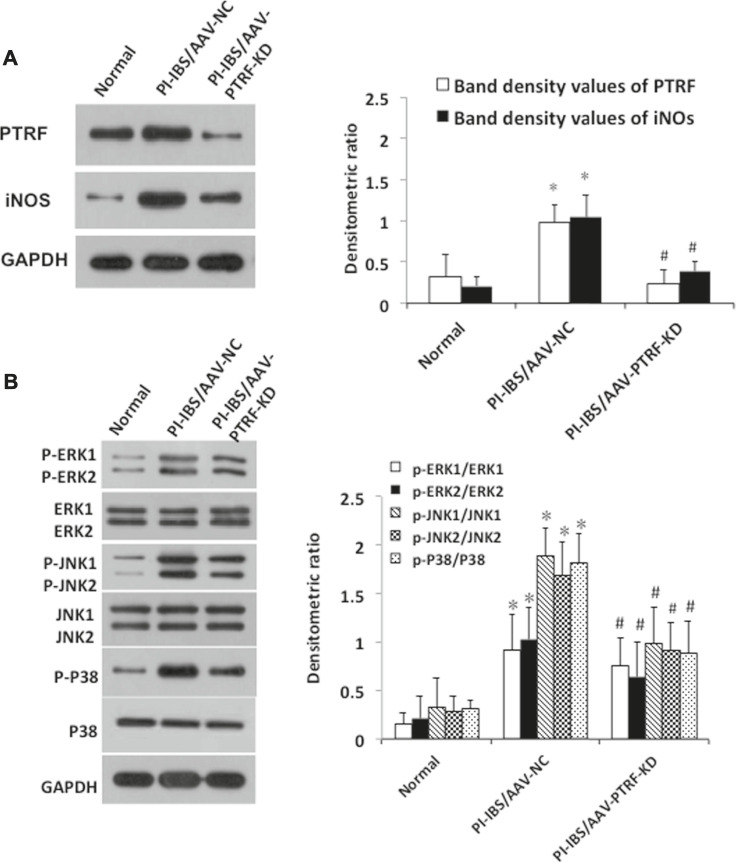
AAV-mediated PTRF knockdown affected TLR4 downstream signaling. **(A)** Homogenates of colons were blotted with anti-PTRF and anti-iNOs. The relative expressions were standardized to the endogenous control GAPDH. **Left:** Representative images of PTRF and iNOs expression; an antibody for GAPDH was used to show equal protein loading; **right:** bar graphs show quantitative evaluation (*n* = 3). Data are reported as the mean ± S.D., *p < 0.05 compared to normal. #*p* < 0.05 compared to PI-IBS-AAV-NC. **(B)** Colonic homogenates were blotted with anti-p38/phospho-p38, anti-ERK/p-ERK, and anti-JNK/p-JNK. The relative expressions were standardized to the endogenous control GAPDH. **Left:** representative images of p38/phospho-p38, ERK/p-ERK, and JNK/p-JNK blots; an antibody for GAPDH was used to show equal protein loading; **right:** bar graphs show quantitative evaluation (*n* = 3). Data are reported as the mean ± S.D., *p < 0.05 compared to normal. #*p* < 0.05 compared to PI-IBS-AAV-NC.

Since *in vitro* silencing of PTRF significantly inhibited LPS-induced inflammation, we investigated whether the inhibition of PTRF targets could effectively inhibit PI-IBS *in vivo*. To determine intestinal efficiency *in vivo*, WB and IHC assays were used to detect PTRF expression in the intestines (Figures 4, 5). WB analysis indicated that PTRF expression was significantly increased after EPSD, TNBS, and/or CUMS stimulation (Figure 4A).

Recombinant AAV vector-mediated gene delivery to intestinal epithelial cells provides a new approach for gut transduction and allows the study of intestinal diseases (Fang et al., 2019). According to a previous study, we selected AAV serotype 9, which provides a relatively high efficiency in gut transduction (Polyak et al., 2012) and transfected rats with AAV-PTRF knockdown (AAV-PTRF-KD) or control (AAV-NC) vectors through tail vein injection. Luciferase activity was quantified as a reporter in this study. At day 1, animals treated with AAV vectors containing the luciferase sequence demonstrated positive luciferase expression, which peaked at week 12 (Figure 4D). We showed that AAV-PTRF-KD led to a reduction in PTRF protein levels compared with AAV-NC in targeted regions (colons) in PI-IBS rats (Figures 4B, 5A).

To determine whether PTRF was localized with TLR4, we co-stained the tissue slides with anti-PTRF and anti-TLR4 antibodies. We found a strong co-localization pattern for PTRF and TLR4. Furthermore, TLR4/PTRF co-expression decreased when PTRF was knocked down by AAV-PTRF shRNA (Figure 4C). Consequently, the activation of TLR4 downstream signaling, including the p38, ERK, and JNK pathways, was significantly suppressed by AAV-PTRF-KD in PI-IBS rats. As shown in Figure 5A, PI-IBS-induced expression of iNOs and generation of NO were markedly diminished in the PI-IBS-AAV-PTRF-KD group compared with those in the control group.

### AAV-Mediated PTRF Knockdown Alleviates Inflammation and Ameliorates Body Weight, Sucrose Intake, and Fecal Water Content in PI-IBS Rats

**FIGURE 6:**
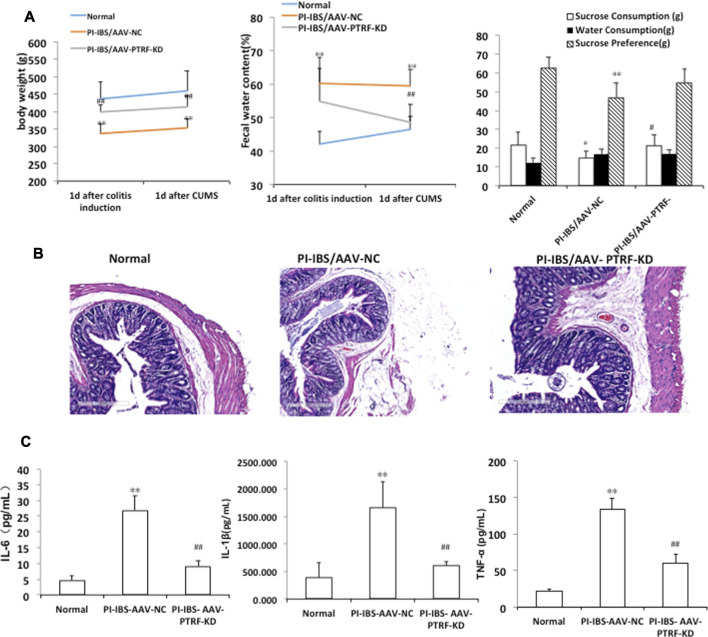
AAV-mediated PTRF knockdown alleviates PI-IBS in rats. **(A)** **Left**, rat’s body weight; **middle**, fecal water content; and **right**, sucrose preference. **(B)** Representative images of H&E staining of colon sections, scale bar = 300 μm **(C)** IL-1β, IL-6, and TNF-α expression in supernatants of intestinal mucosa homogenates. Data are presented as the mean ± SEM, *n* = 6. *p < 0.05, **p < 0.01 compared to normal. ^#^ *p* < 0.05, ^##^ *p* < 0.01 compared to PI-IBS-AAV-NC.

To determine whether PTRF loss contributed to PI-IBS, we performed AAV-mediated genetic knockdown. Body weight, sucrose intake, and fecal pellets were measured to assess the induction of PI-IBS. In addition, the colons of rats were stained with H&E to observe the histological changes. IL-1β, IL-6, and TNF-α levels in colonic homogenates were evaluated in each group. The PI-IBS-AAV-NC group showed a significant decrease in body weight, which coincided with a higher fecal water content (Figure 6A). Figure 6A also shows the volume of water and sucrose consumed during the sucrose preference test. ANOVA revealed that there was a significant decrease in sucrose preference after CUMS treatment. However, AAV-mediated PTRF knockdown increased body weight and sucrose preference, which coincided with the decreased fecal water content. These results suggest that suppression of PTRF ameliorates symptoms of PI-IBS, such as intestinal functions and depression.

H&E staining showed that the colonic epithelial cells in each group were arranged regularly. Cell inflammation, infiltration, or colonic epithelial cell damage, with slight shrinkage and swelling of cellular processes, was observed in the PI-IBS-AAV-NC group. AAV-mediated PTRF knockdown alleviated colonic epithelial cell damage, including inflammation and swelling, in the PI-IBS group. These findings indicate that PTRF contributes to inflammation injury induced by TNBS. Cytokine levels in the colon homogenates, including IL-1β, IL-6, and TNF-α, were significantly higher in the PI-IBS-AAV-NC group compared to those in the normal group and were significantly lower in the PI-IBS-AAV-PTRF-KD group (Adriani et al., 2018).

## Discussion

As a key component of the caveolae structure on the plasma membrane, PTRF has been widely reported as a tumor suppressor (Bai et al., 2012; Gámez-Pozo et al., 2012; Nassar et al., 2013; Moon et al., 2014). On the other hand, several studies have found that PTRF promotes progression and resistance to treatment in breast cancer, pancreatic cancer, glioblastomas, and colorectal cancer (Yi et al., 2013; Wang et al., 2014). Such contradicting observations have led multiple research groups to postulate that the role of PTRF in cancer varies with the specific type and stage (Nassar and Parat, 2015). We studied the function of PTRF in the development of colorectal cancers and reported that the expression of PTRF is significantly reduced in tumor tissues derived from human patients with colorectal cancers. In addition, we found that the expression of PTRF negatively regulates the tumorigenic activities of colorectal cell lines (HcoEpiC, Colo320, HT29, and CaCo_2_). Furthermore, biochemical studies revealed that overexpression of PTRF led to the suppression of the AKT/mTOR pathway, as evidenced by reduced phosphorylation of AKT, mTOR, and downstream MMP-9. Thus, these molecules may serve as potential therapeutic targets for human gastrointestinal diseases (Wang et al., 2017b). However, the function of PTRF in intestinal epithelial cells and gastrointestinal disorders has been disputed. Thus, future studies are needed to define the role of PTRF in the regulation of intestinal epithelial cell functions and functional disorders.

As mentioned above, in our previous study, we found the highest expression of PTRF in the intestinal epithelial cells HCoEpiCs, which are able to trigger an immune response through the innate immune system (Wang et al., 2017b; Sina et al., 2020). PTRF/Cavin-1 regulates LPS-induced inflammation in HCoEpiCs, as indicated by the phosphorylation of MAPK signaling cascades and iNOS demonstrated in cellular experiments. PTRF has numerous functions, including cell signaling and lipid regulation (Hill et al., 2008). The MAPK signaling pathway mediates many pathological responses, including inflammation and apoptosis (Dong et al., 2020). Specific signaling pathways associated with inflammation, such as MAPK pathways, tyrosine kinases, and eNOS, are regulated by caveolin and bind to pro-survival and pro-growth molecules (Tian et al., 2020). In addition, caveolin protein regulation of eNOS modulates inflammatory signaling via the local control of NO production (Dong et al., 2020). In this study, we reported that PTRF deletion in HCoEpiCs reduced iNOS expression and significantly inhibited the phosphorylation of ERK, p38, and JNK, suggesting that PTRF indeed regulates LPS-induced inflammation. Moreover, investigating whether inhibition of PTRF targets could effectively induce similar effects *in vivo*, we performed AAV-mediated PTRF knockdown in PI-IBS rats, which resulted in suppressed p38, ERK, and JNK pathway activation. These findings suggested that PTRF ameliorated inflammation through the regulation of MAPK and iNOS pathways.

Evidence supports that TLR4 is a key receptor that provides a rapid response in gut innate immunity, especially in intestinal inflammation (Hausmann et al., 2002; Wang et al., 2015a). The mechanism derived from LPS-induced inflammation is mainly attributed to the suppression of MAPK signaling cascades by the inhibition of TLR4 signaling (Wang et al., 2017c). Signaling cascades (TLR2/6, TLR4, and TLR9) have been shown to converge with the signaling pathway of the anaphylatoxin receptors C3aR and C5aR1 at the MAPKs, primarily ERK1/2 and JNK, resulting in the increase of pro-inflammatory cytokines including TNF, IL-1β, and IL-6 (Sina et al., 2020). Moreover, the reduction of PTRF resulted in its interaction with TLR4 and regulation of the TLR4 signaling pathway. Moreover, caveolae have been found to be the receptor of downstream molecules (Liu et al., 2019). Additionally, studies indicate a PTRF-impaired formation of the TLR4/Myd88 complex, while LPS strengthened the co-localization and interaction between PTRF and TLR4 in lipid rafts (Zheng et al., 2013). Further studies indicate that upon LPS being recognized, TLR4 and its downstream components traffic from the nonraft portion to the lipid raft portion on the plasma membrane. It means that PTRF is required for the TLR4 signaling assembly, especially after LPS stimulation, and helps to “hold” the TLR4 pathway components together in the rafts, which trigger the downstream signal cascades, including ERK, p38, and JNK (Zheng et al., 2013).

In this context, AAV-mediated PTRF knockdown resulted in a reduced TLR4/PTRF interaction, indicating a strong co-localization pattern for PTRF and TLR4. Furthermore, TLR4/PTRF co-expression decreased when PTRF was knocked down by AAV-PTRF shRNA in PI-IBS rats. In fact, the downregulation of PTRF affects all MAPK pathways, including ERK, p38, and JNK (Figure 5). This result shows that the key step of PTRF regulation falls on the initial TLR4 signaling. Thus, it is easily understood that the downregulation of PTRF attenuates TLR4 downstream products, including NO production, iNOS synthesis, and cytokines release. These findings are consistent with the previous reports indicating that a close contact between TLR4 and PTRF is a requisite for restoring intestinal inflammation.

The PI-IBS model rats in our experiments showed greater levels of tension and anxiety, among other emotions. The body weight of these animals decreased with the increased fecal water content, and there was a significant decrease in sucrose preference after EPSD treatment, which is consistent with the results of previous studies (Yu et al., 2021). AAV-mediated PTRF knockdown increased the body weight and sucrose preference, which coincided with the decreased fecal water content. However, a previous study demonstrated that homozygous PTRF knockout mice had a leaner body mass compared to wild-type animals (Low et al., 2014). This contradiction could be attributed to comprehensive factors including the modeling method, research purposes, species of animals, etc. As reported previously, IBS patients have distinctly increased serum TNF-α and IL-1β levels. Particularly, the IL-1β level is increased in PI-IBS patients (Ortiz Lucas et al., 2010). Equally, serum cytokine levels, including IL-1β, IL-6, and TNF-α, were significantly higher in the PI-IBS-AAV-NC group compared with those in the normal group. Cell inflammation, infiltration, or colonic epithelial cell damage, slightly shrunken and surrounded by swollen cellular processes, was observed in the PI-IBS-AAV-NC group. PTRF-knockdown treatment significantly attenuated inflammation. Overall, these results suggest that suppression of PTRF ameliorates symptoms of PI-IBS such as intestinal functions and depression.

## Conclusion

In conclusion, PTRF has been demonstrated to attenuate symptoms of PI-IBS, such as the inflammation process, both *in vitro* and *in vivo* by regulating the expression of PTRF depending on the phosphorylation of MAPK signaling cascades and iNOS, affecting TLR4/PTRF co-expression and ameliorating tension emotions. These results imply that PTRF could be used as a target for therapeutic interventions for functional gastrointestinal disorders.

## Data Availability

The original contributions presented in the study are included in the article/Supplementary Material; further inquiries can be directed to the corresponding authors.
