# Supplementary figures and images for: Suppression of PTRF Alleviates Post-Infectious Irritable Bowel Syndrome via Downregulation of the TLR4 Pathway in Rats
Source: Front Pharmacol. 2021 Oct 7;12:724410. doi: 10.3389/fphar.2021.724410 (PMC8529073; doi:10.3389/fphar.2021.724410)

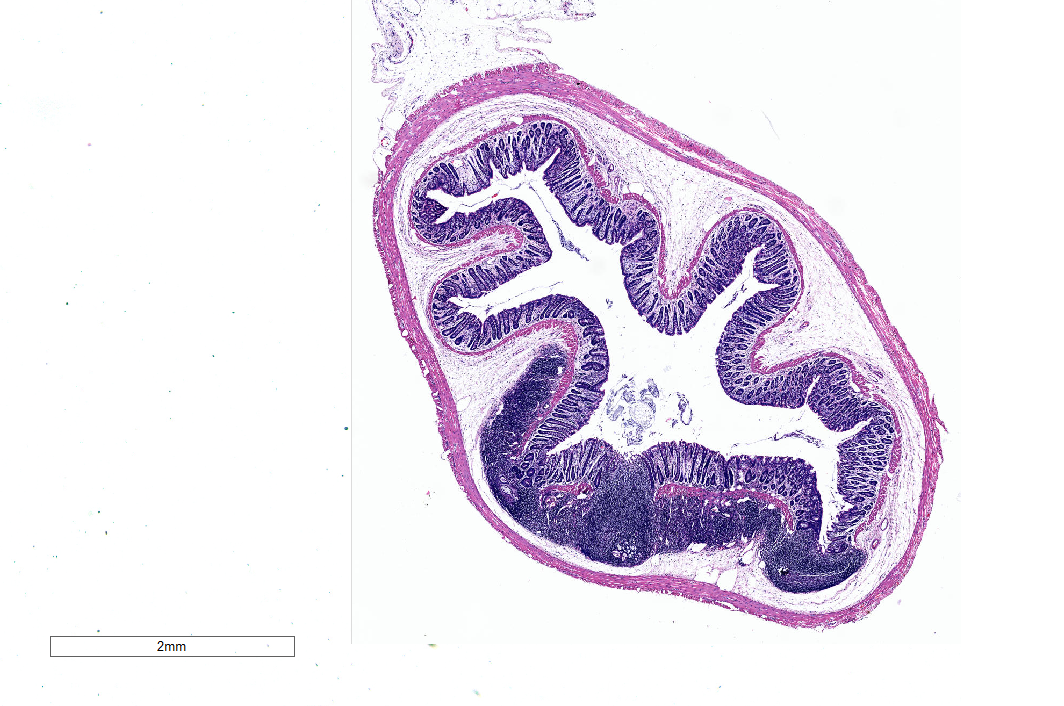

Supplement: Supplementary file 3 [file DataSheet3.ZIP › HE /∩╝êFigure 6 B∩╝ë PI-IBS-KD.tif]

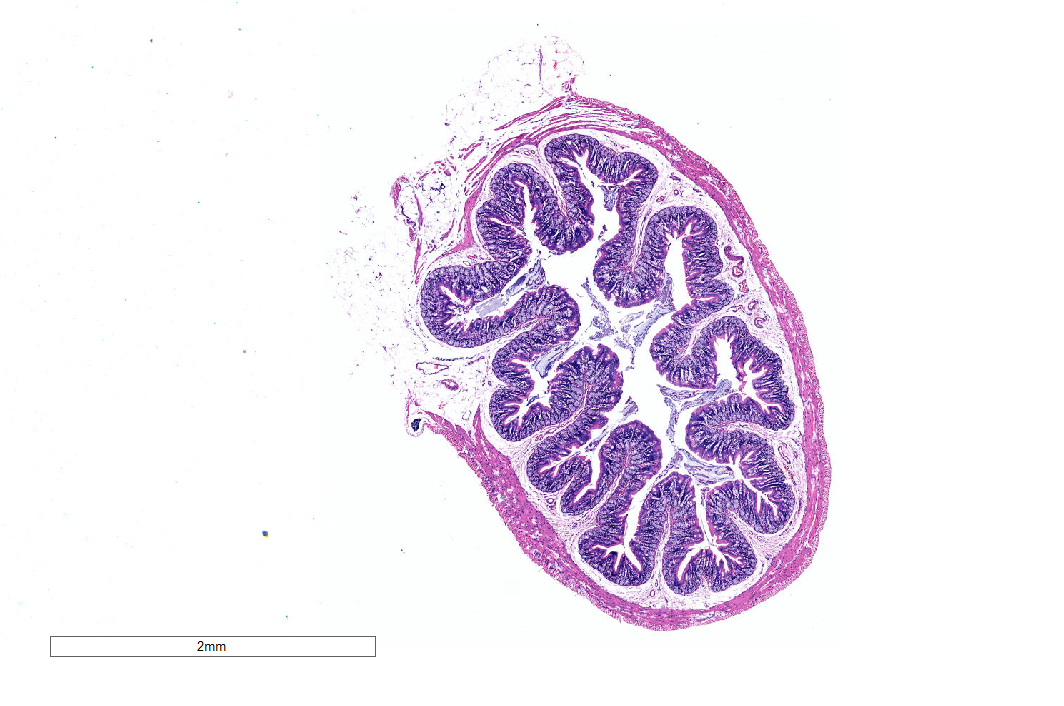

Supplement: Supplementary file 3 [file DataSheet3.ZIP › HE /(Figure 6B) PI-IBS-NC.tif]

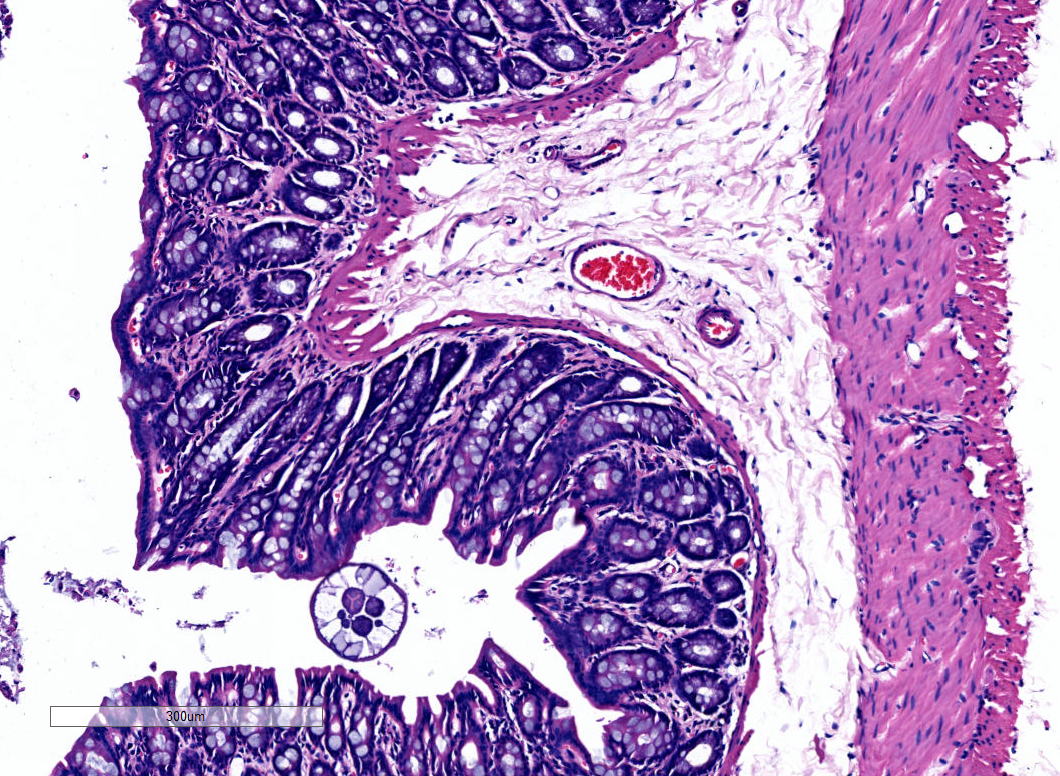

Supplement: Supplementary file 3 [file DataSheet3.ZIP › HE /∩╝êFigure 6 B∩╝ë PI-IBS-KD-H-2.tif]

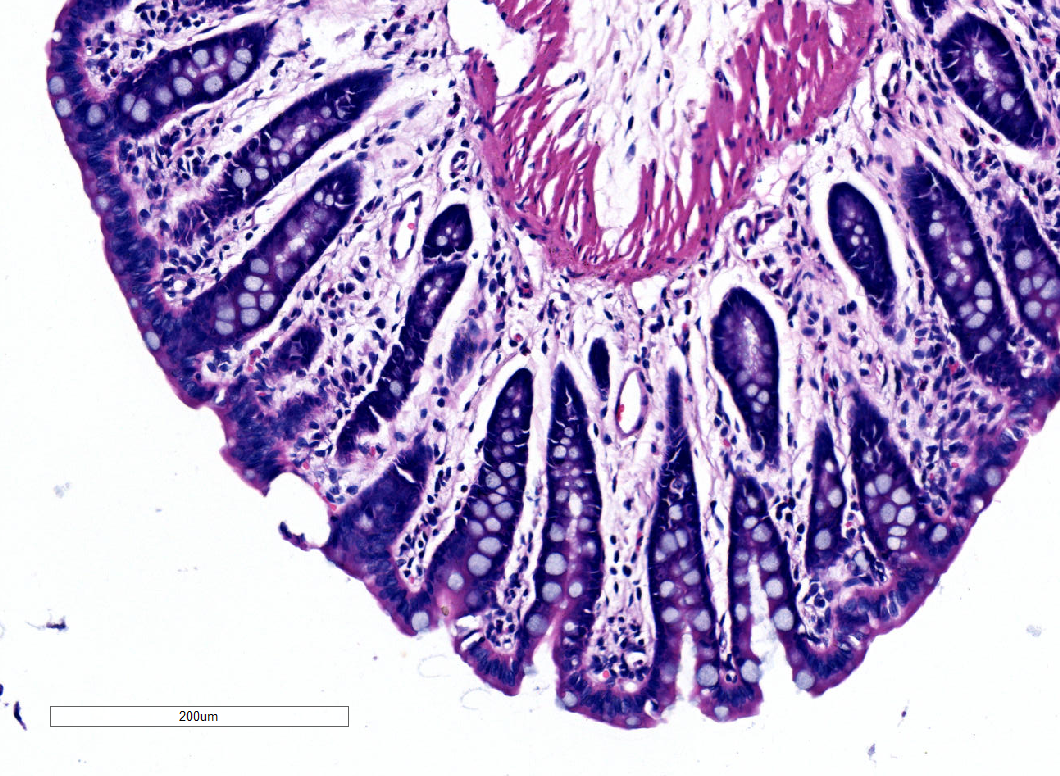

Supplement: Supplementary file 3 [file DataSheet3.ZIP › HE /∩╝êFigure 6 B∩╝ë PI-IBS-KD-H-2 (10bei).tif]

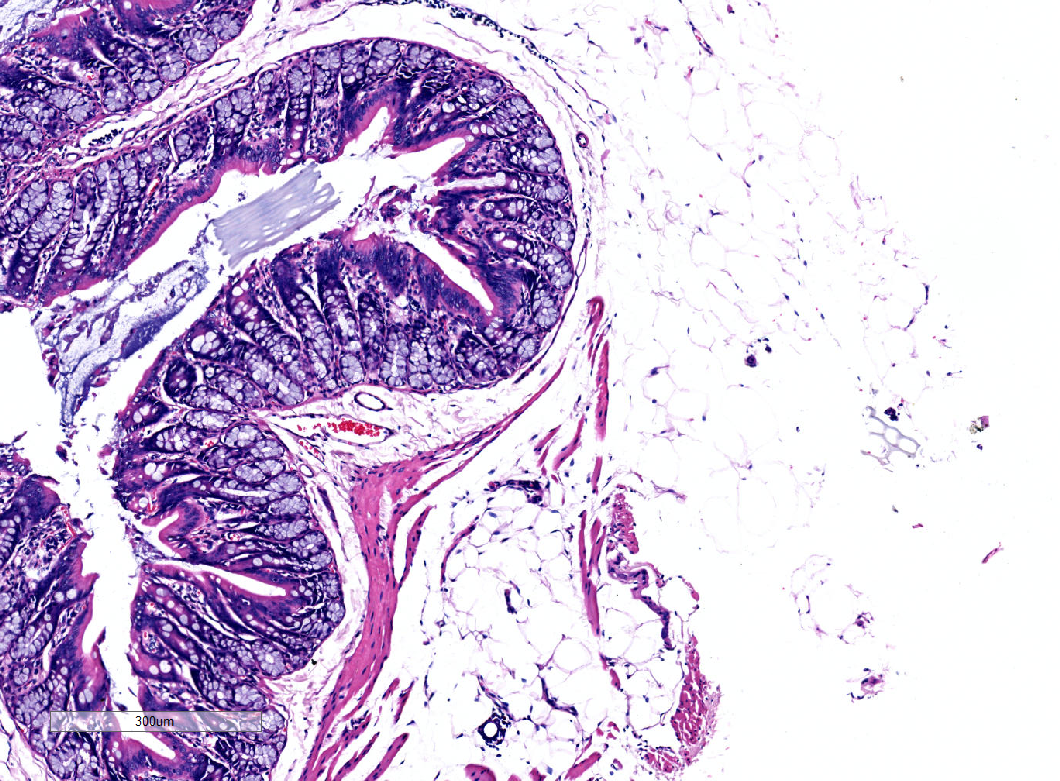

Supplement: Supplementary file 3 [file DataSheet3.ZIP › HE /(Figure 6B)PI-IBS-NC-H-5.tif]

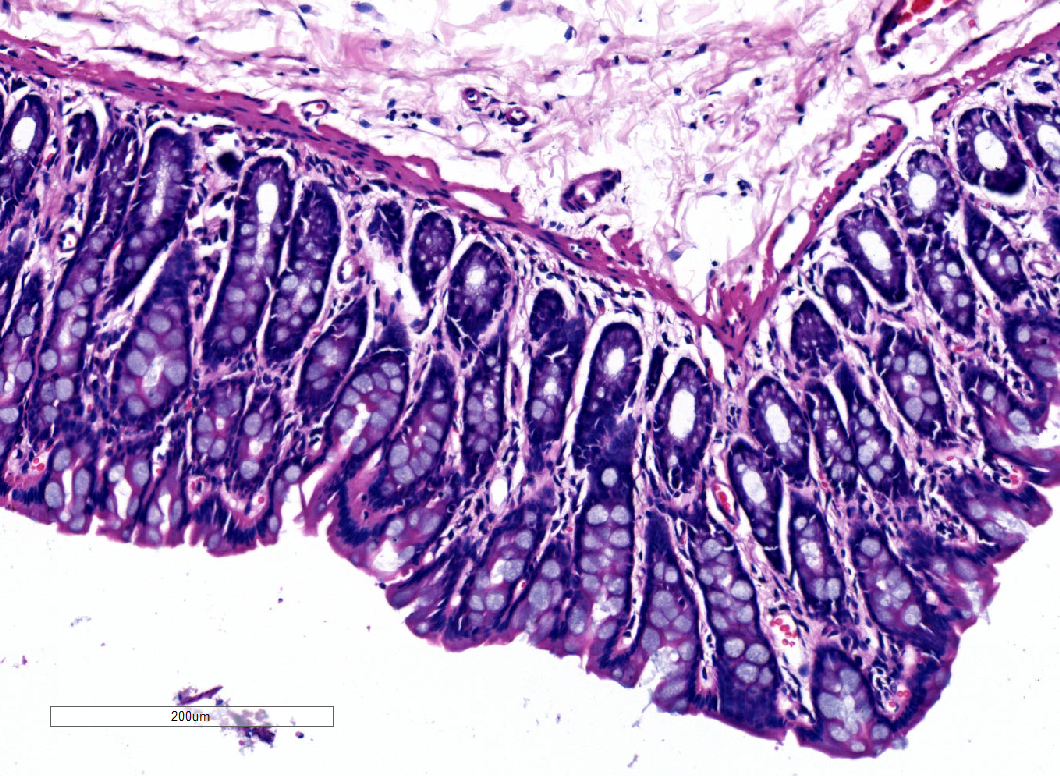

Supplement: Supplementary file 3 [file DataSheet3.ZIP › HE /(Figure 6B)N-H-2(10 bei).tif]

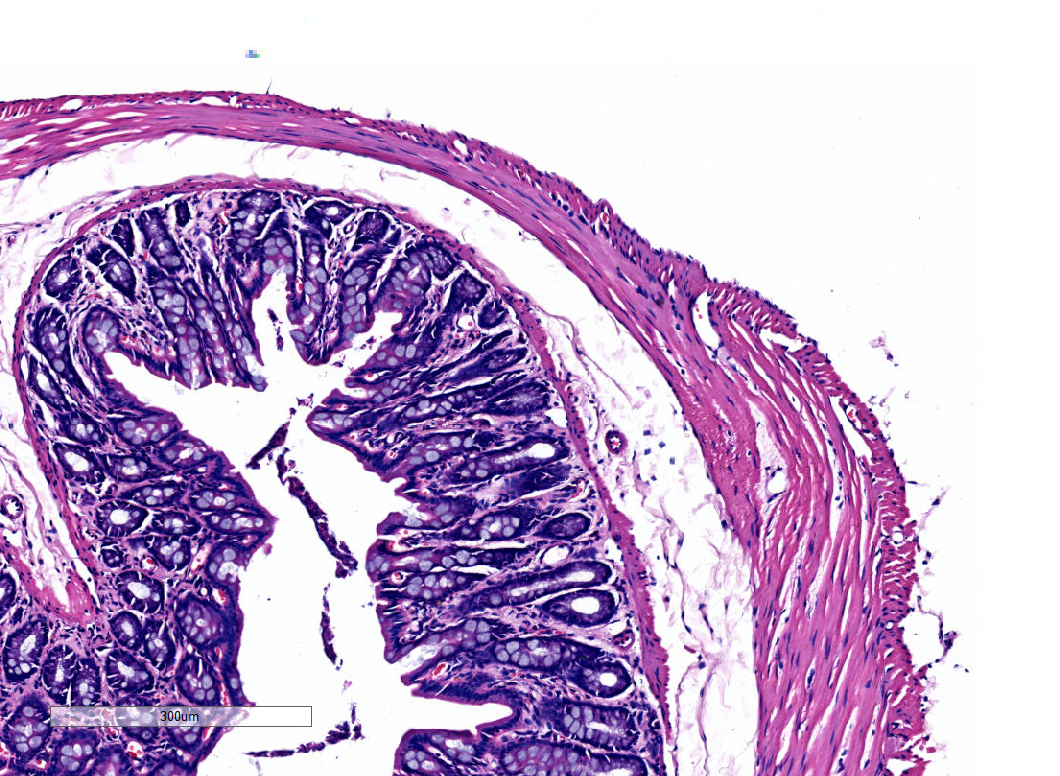

Supplement: Supplementary file 3 [file DataSheet3.ZIP › HE /(Figure 6B) N-H-2.tif]

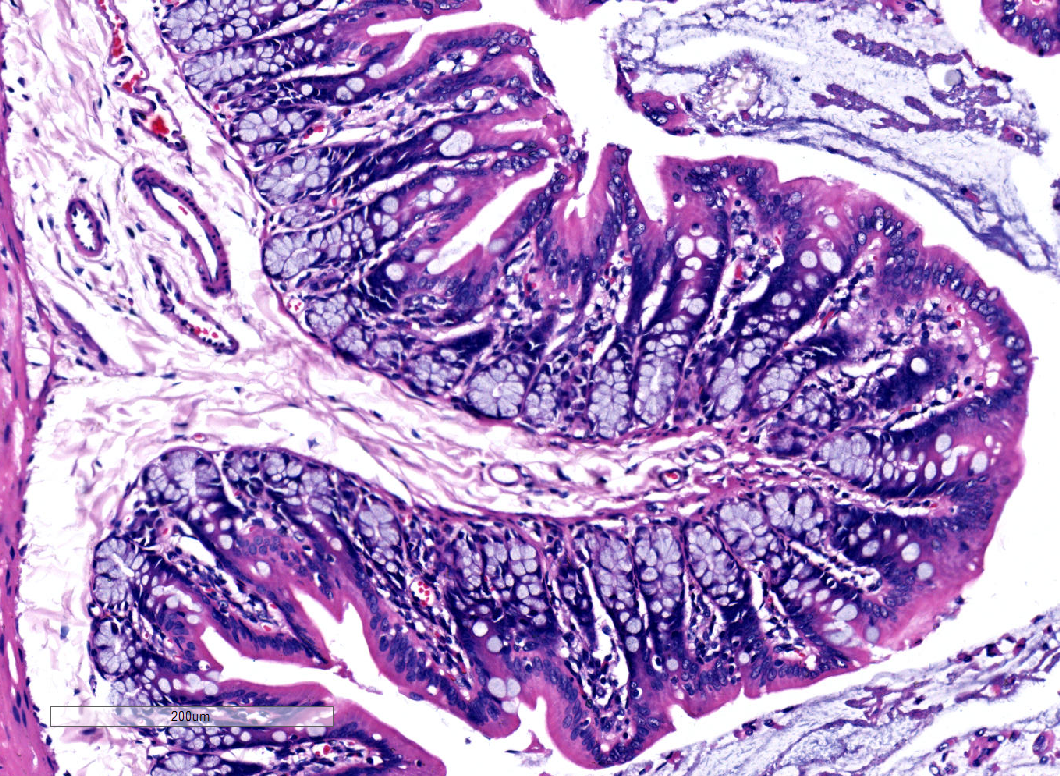

Supplement: Supplementary file 3 [file DataSheet3.ZIP › HE /(Figure 6B)PI-IBS-NC-H-5 (10σÇì∩╝ë.tif]

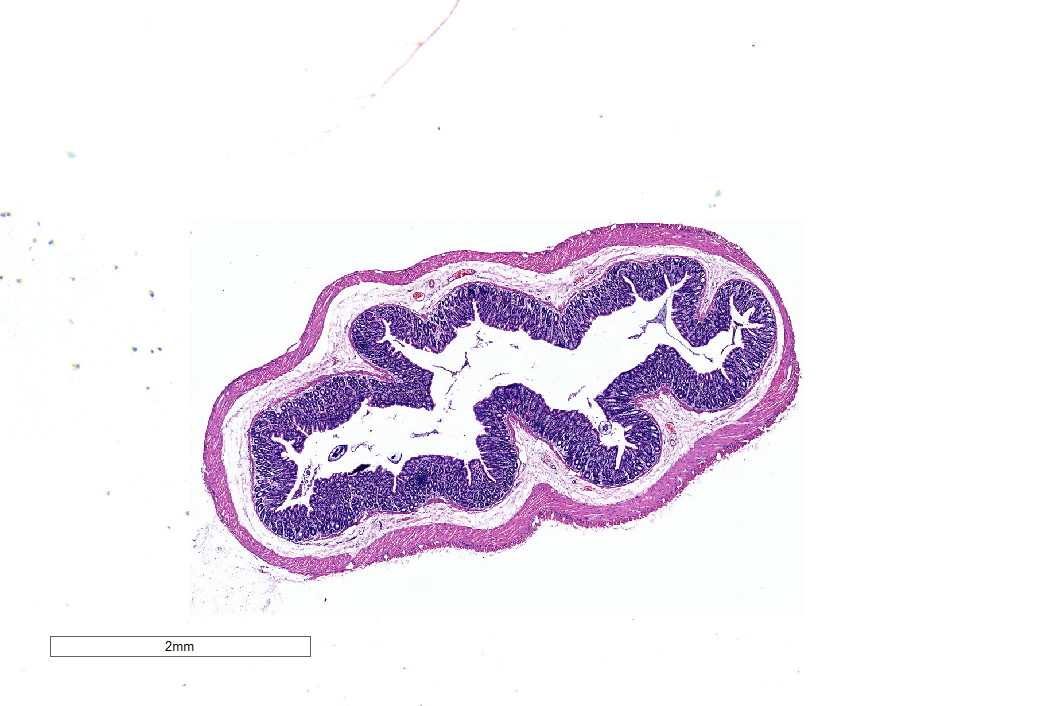

Supplement: Supplementary file 3 [file DataSheet3.ZIP › HE /(Figure 6B)N.tif]

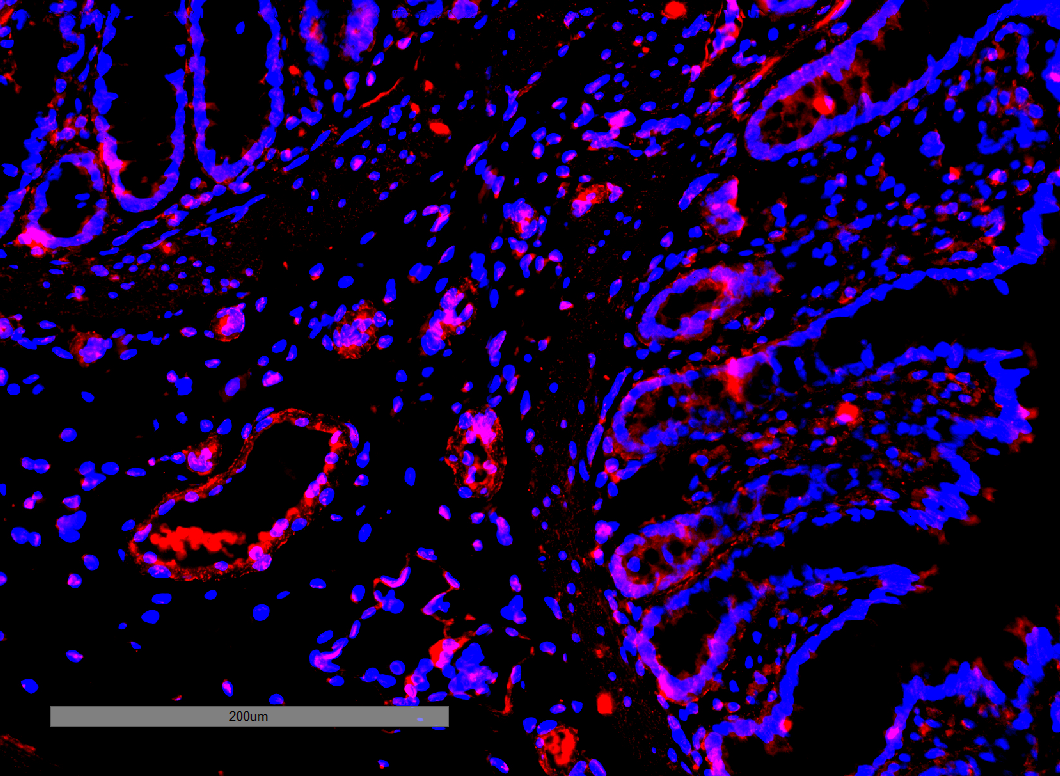

Supplement: Supplementary file 4 [file Image6.TIF]

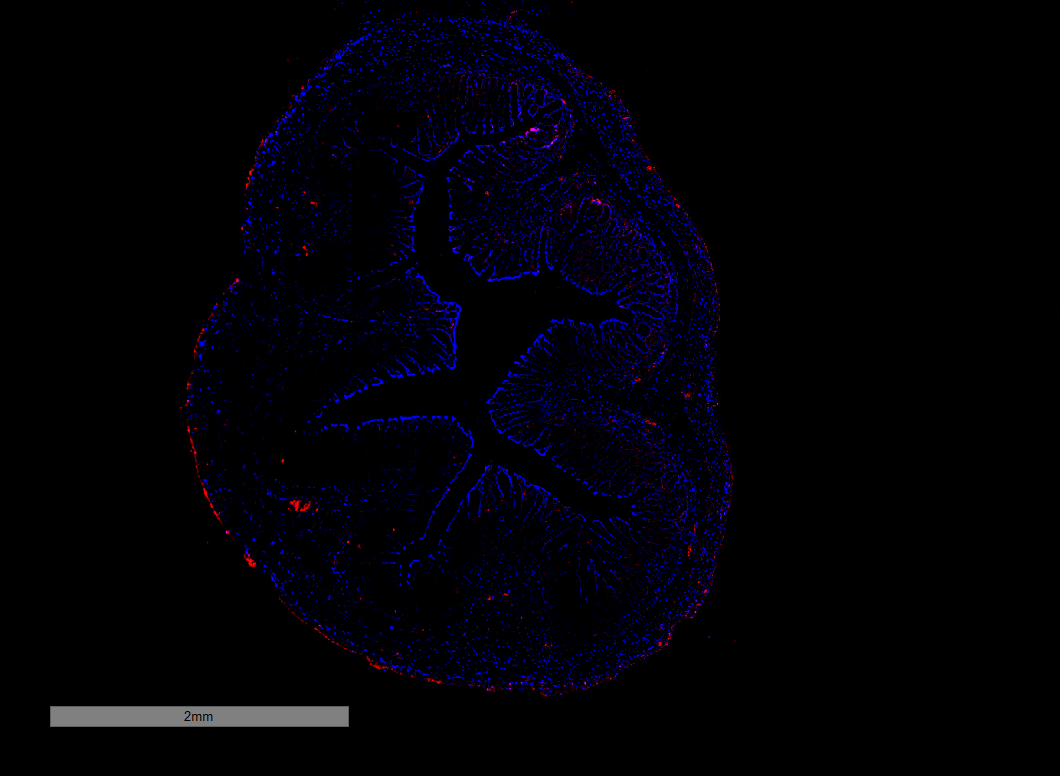

Supplement: Supplementary file 5 [file Image3.TIF]

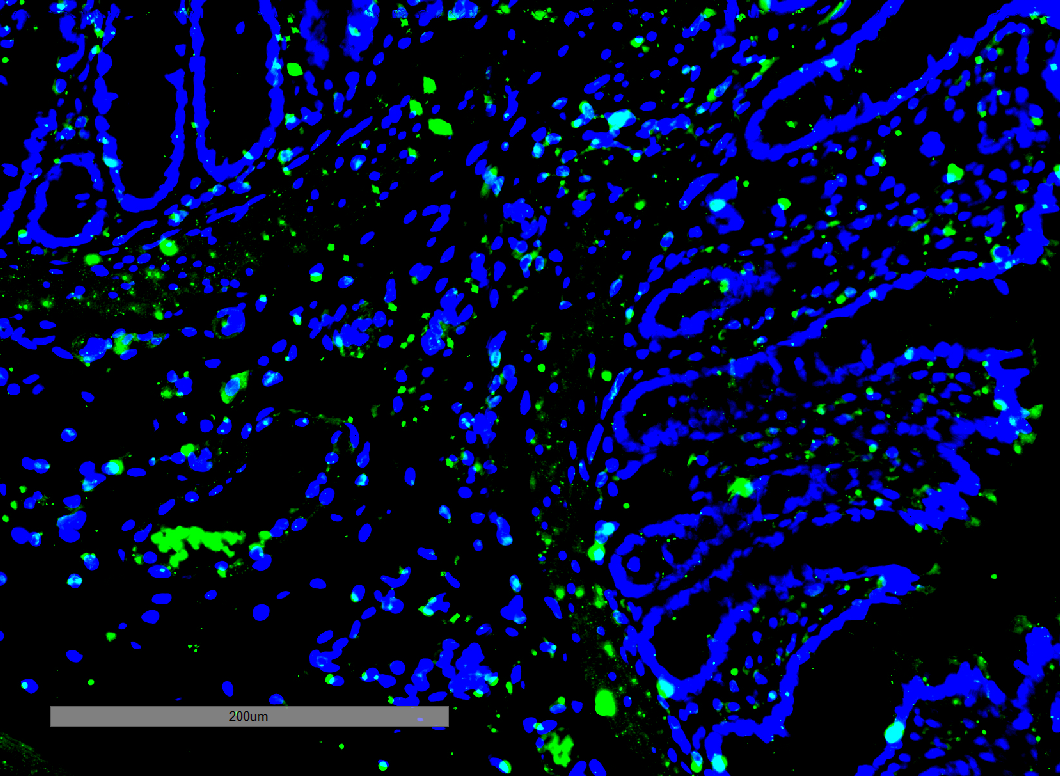

Supplement: Supplementary file 6 [file Image4.TIF]

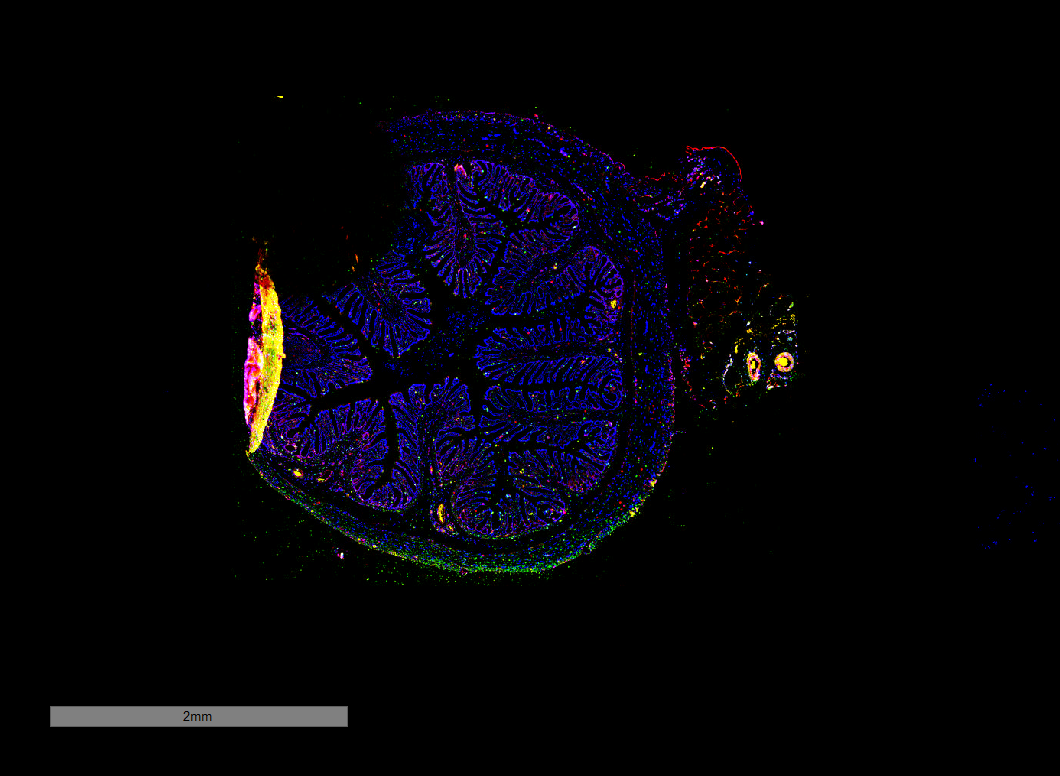

Supplement: Supplementary file 7 [file Image9.TIF]

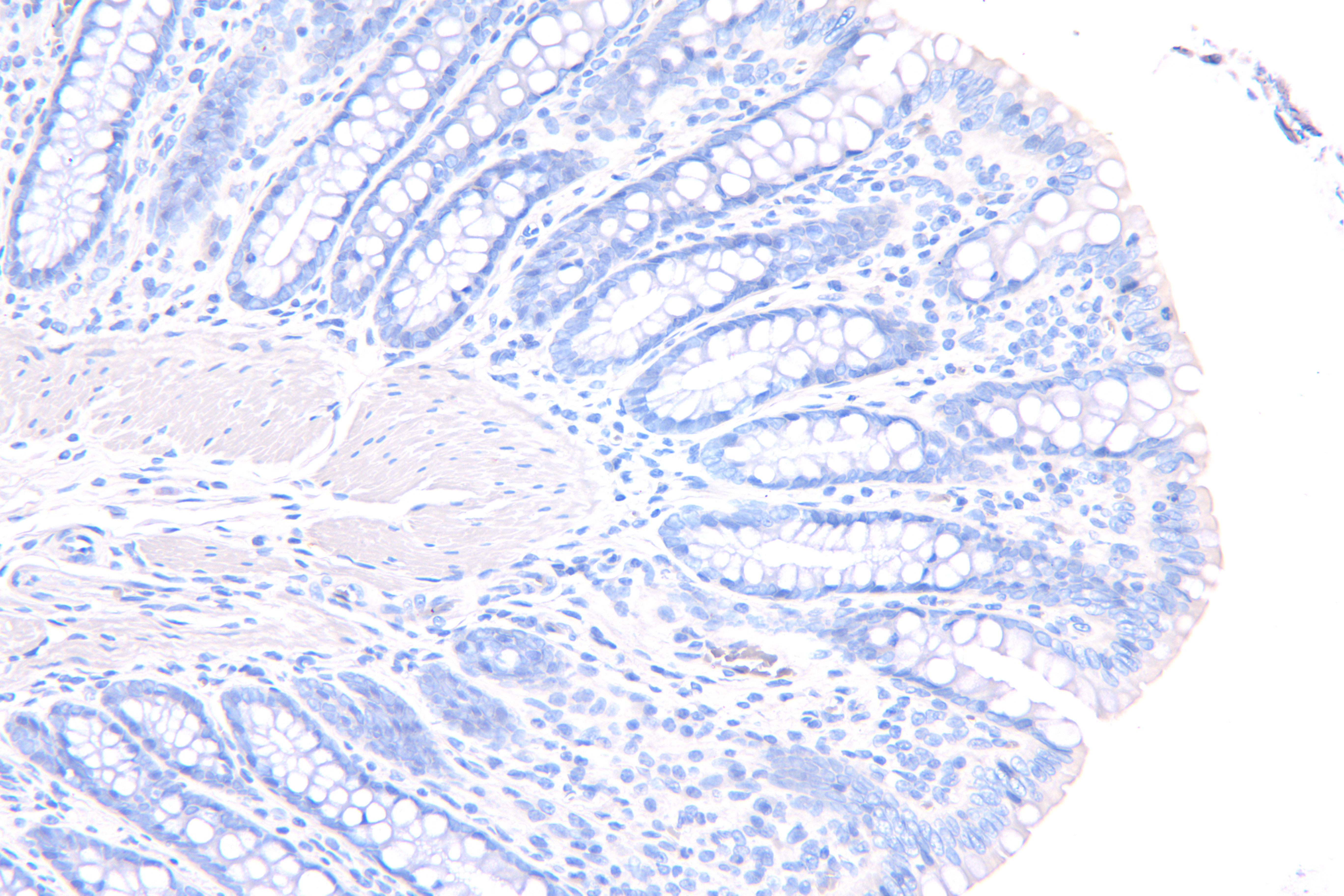

Supplement: Supplementary file 8 [file DataSheet1.ZIP › IHC/Figure 4 A(1).jpeg]

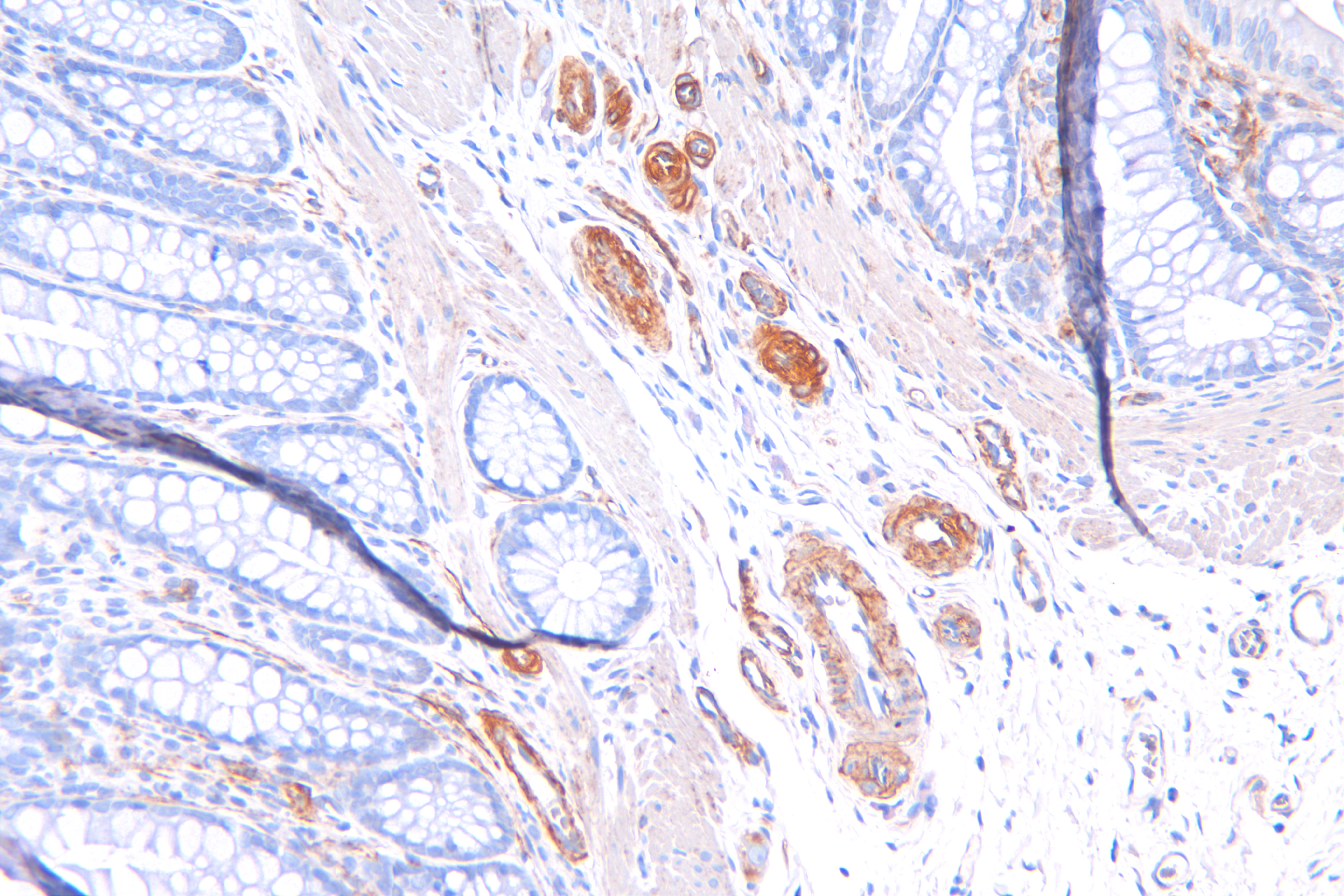

Supplement: Supplementary file 8 [file DataSheet1.ZIP › IHC/Figure4 A(2).jpeg]

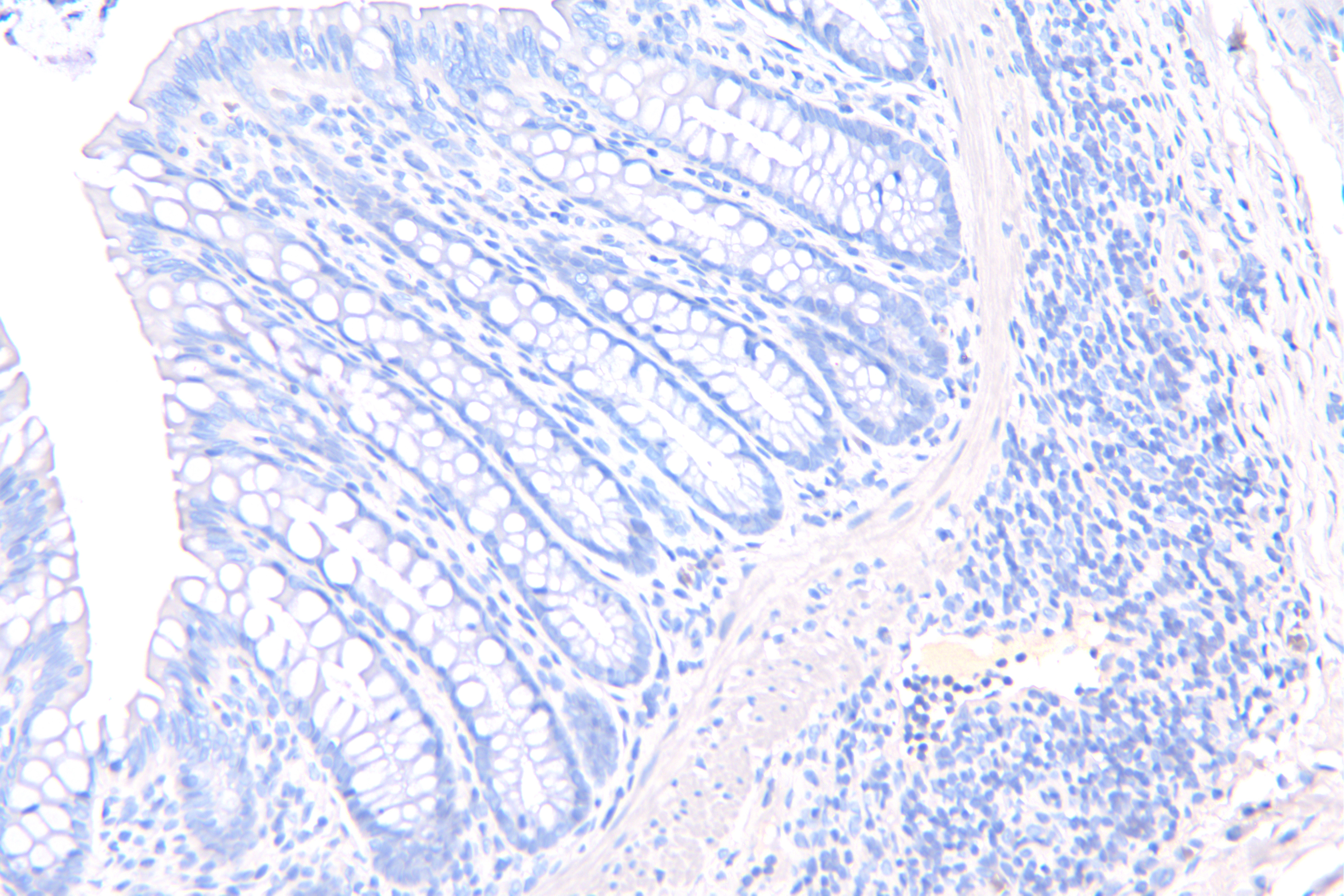

Supplement: Supplementary file 8 [file DataSheet1.ZIP › IHC/Figure 4 A (3∩╝ë.jpeg]

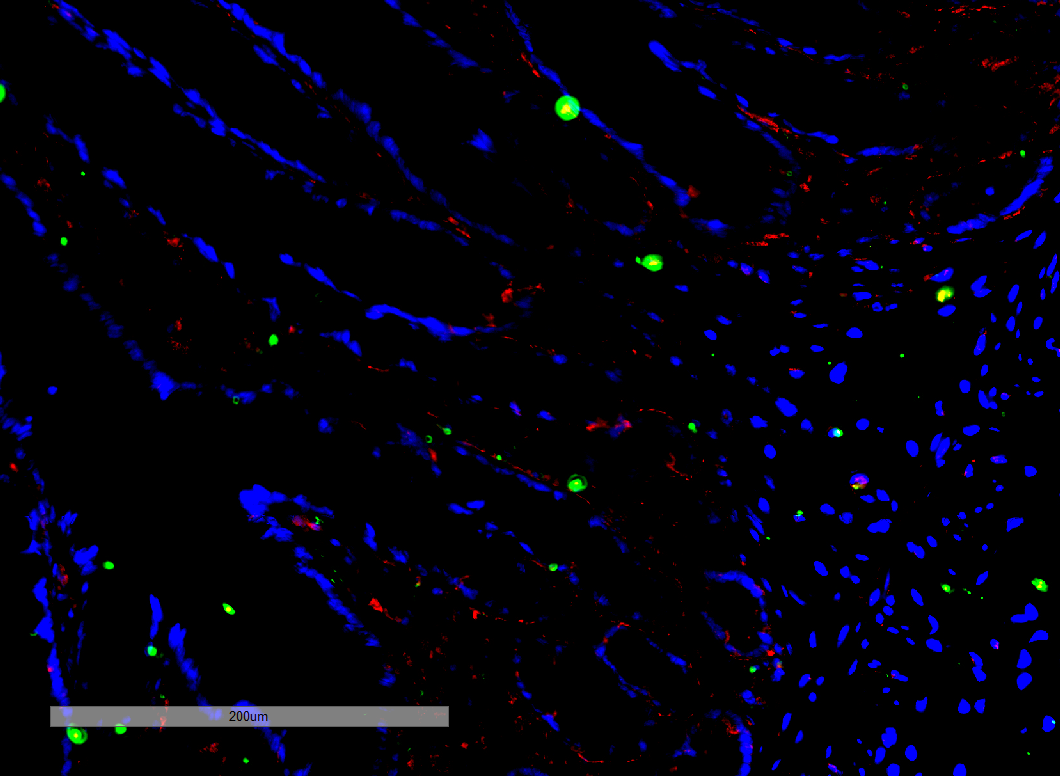

Supplement: Supplementary file 9 [file Image2.TIF]

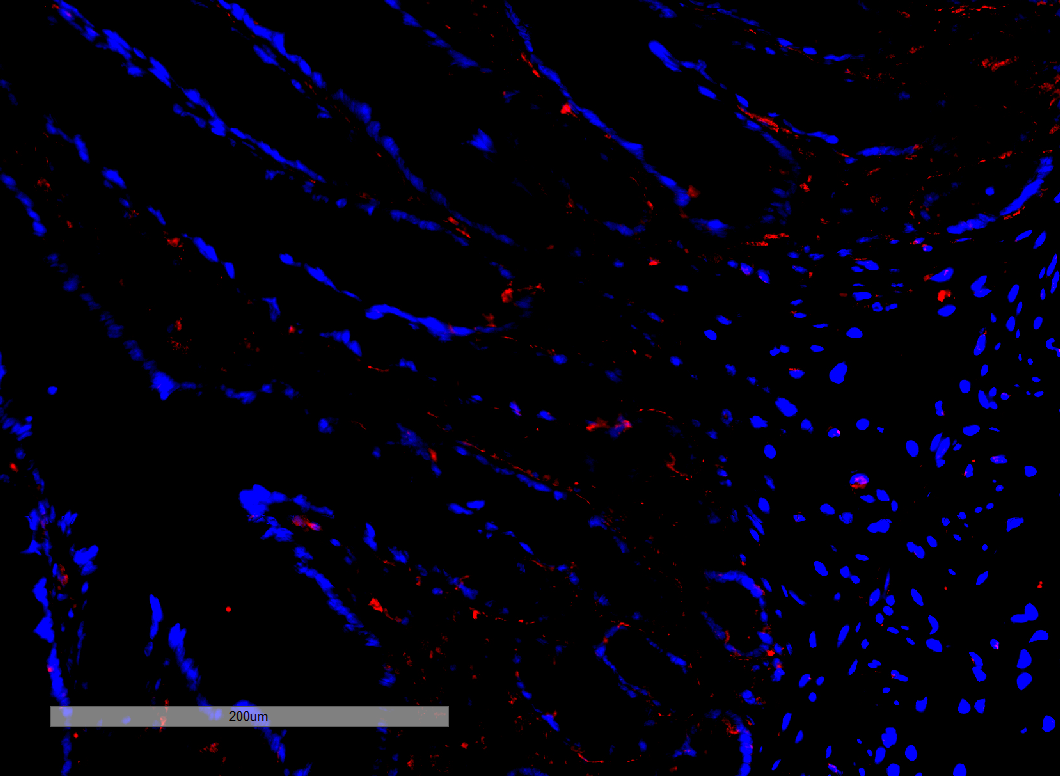

Supplement: Supplementary file 10 [file Image11.TIF]

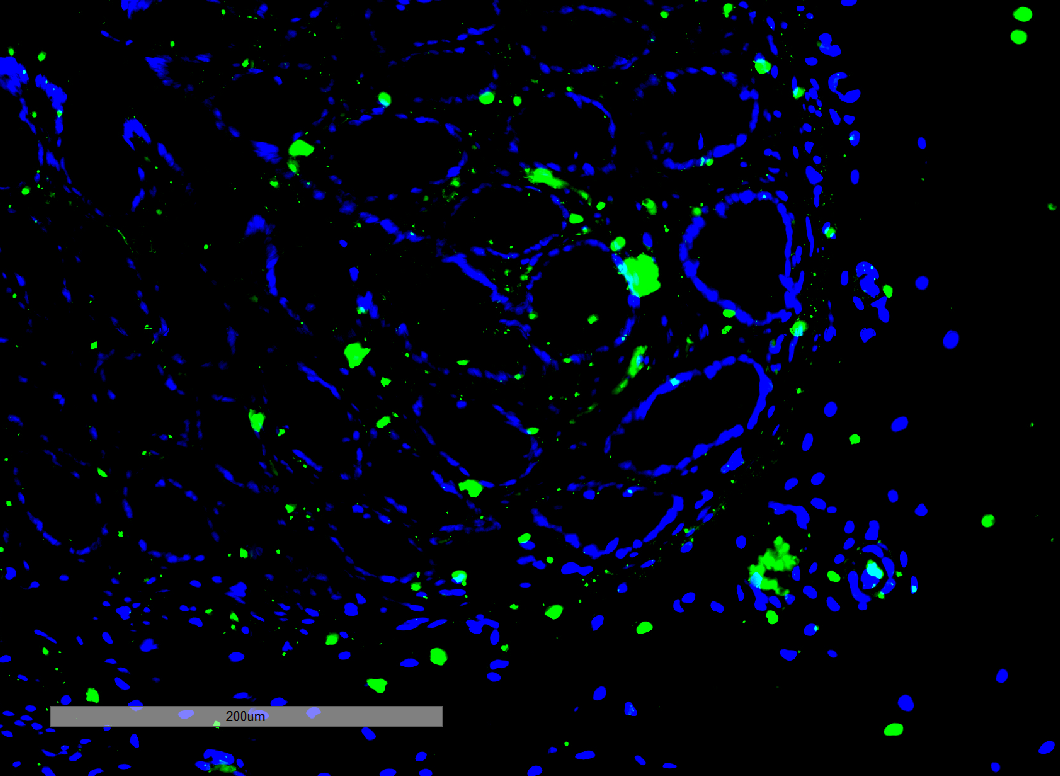

Supplement: Supplementary file 11 [file Image1.TIF]

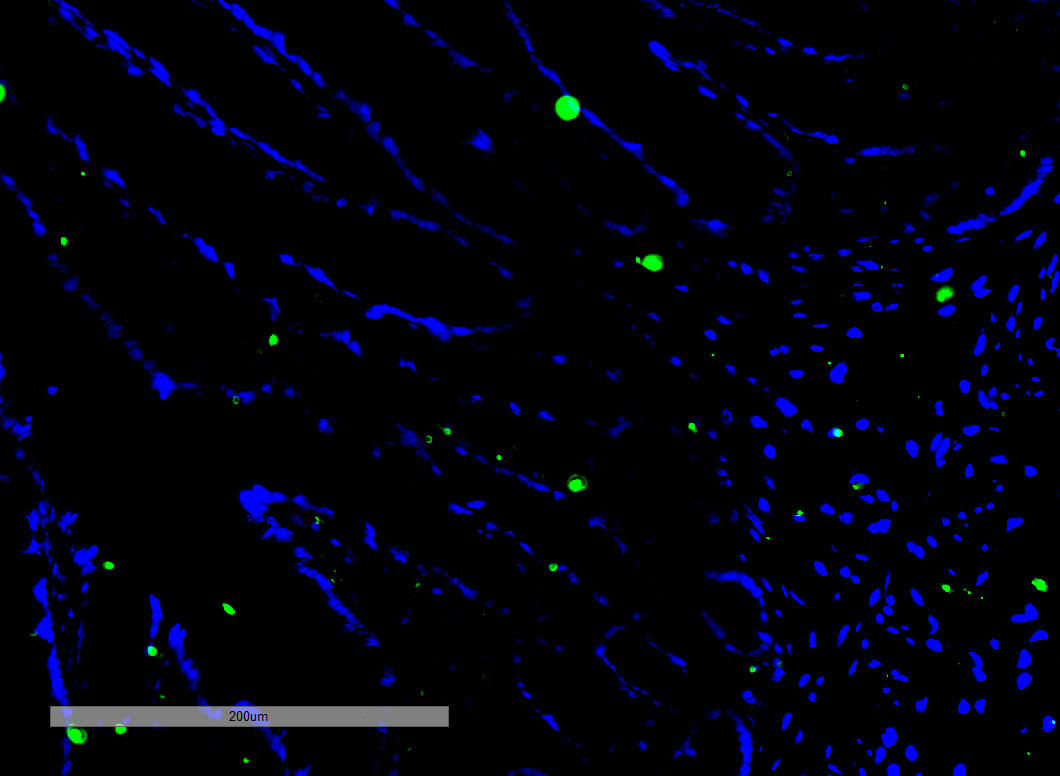

Supplement: Supplementary file 12 [file Image10.TIF]

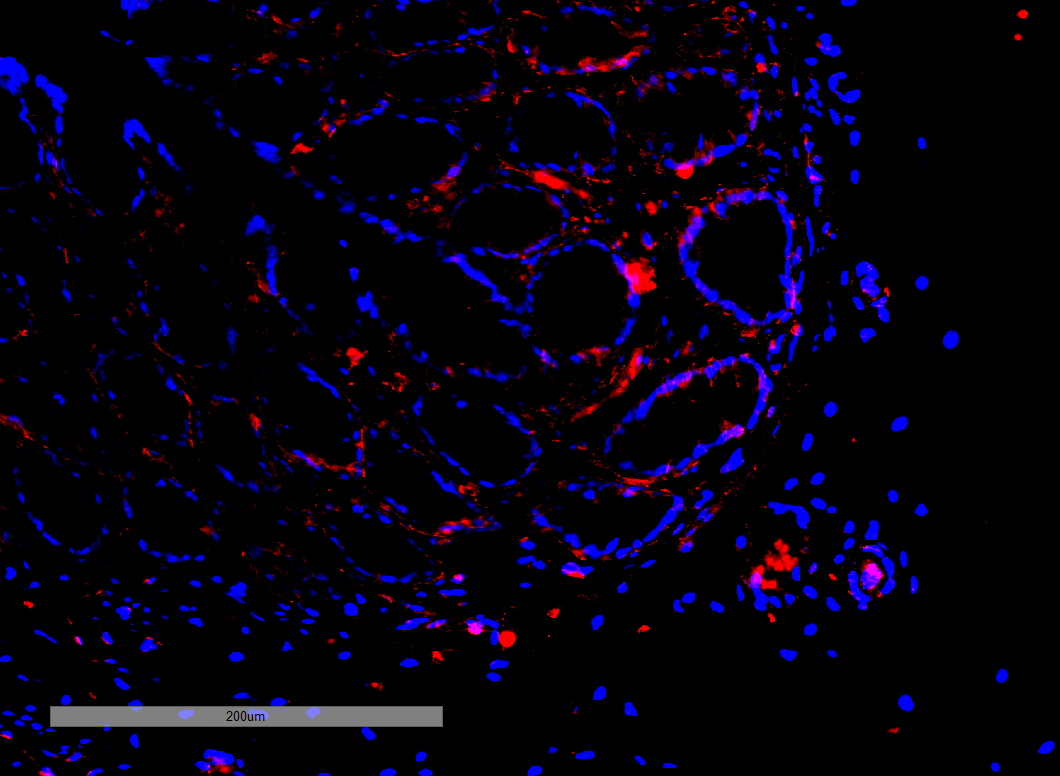

Supplement: Supplementary file 13 [file Image7.TIF]

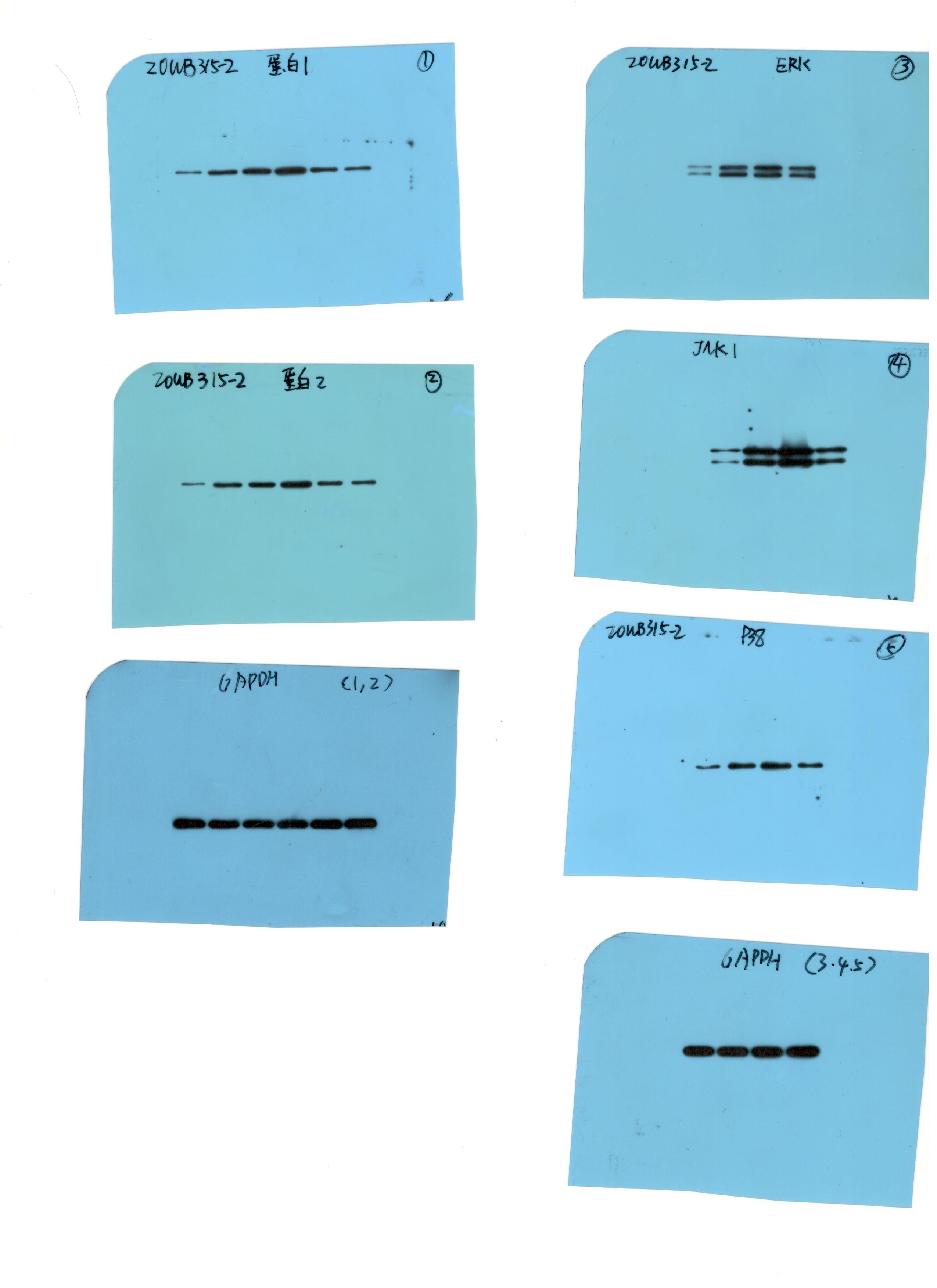

Supplement: Supplementary file 14 [file DataSheet2.ZIP › W-B/Figure 2 western blot∩╝êA∩╝ë.jpg]

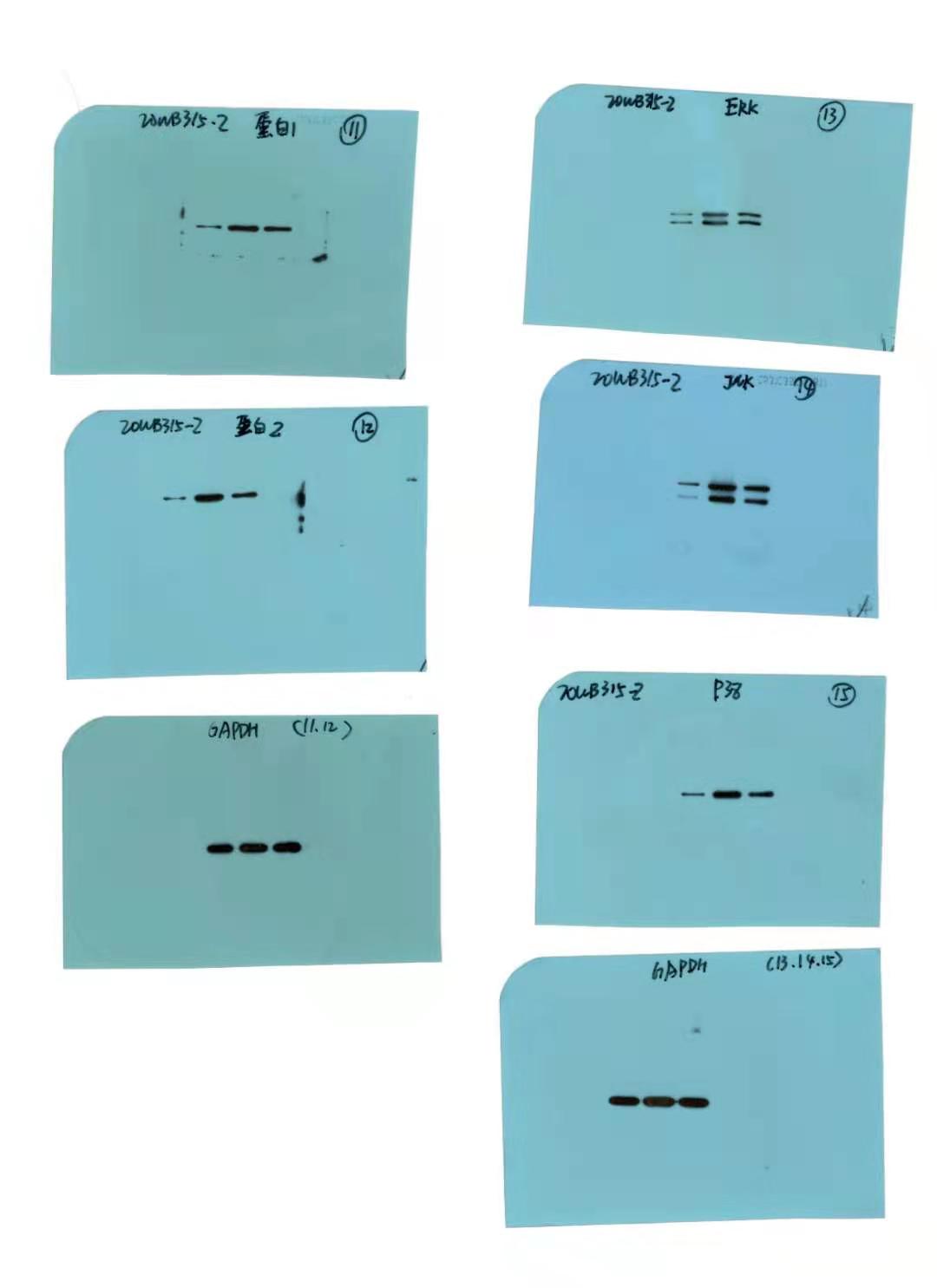

Supplement: Supplementary file 14 [file DataSheet2.ZIP › W-B/Figure 5 western .jpeg]

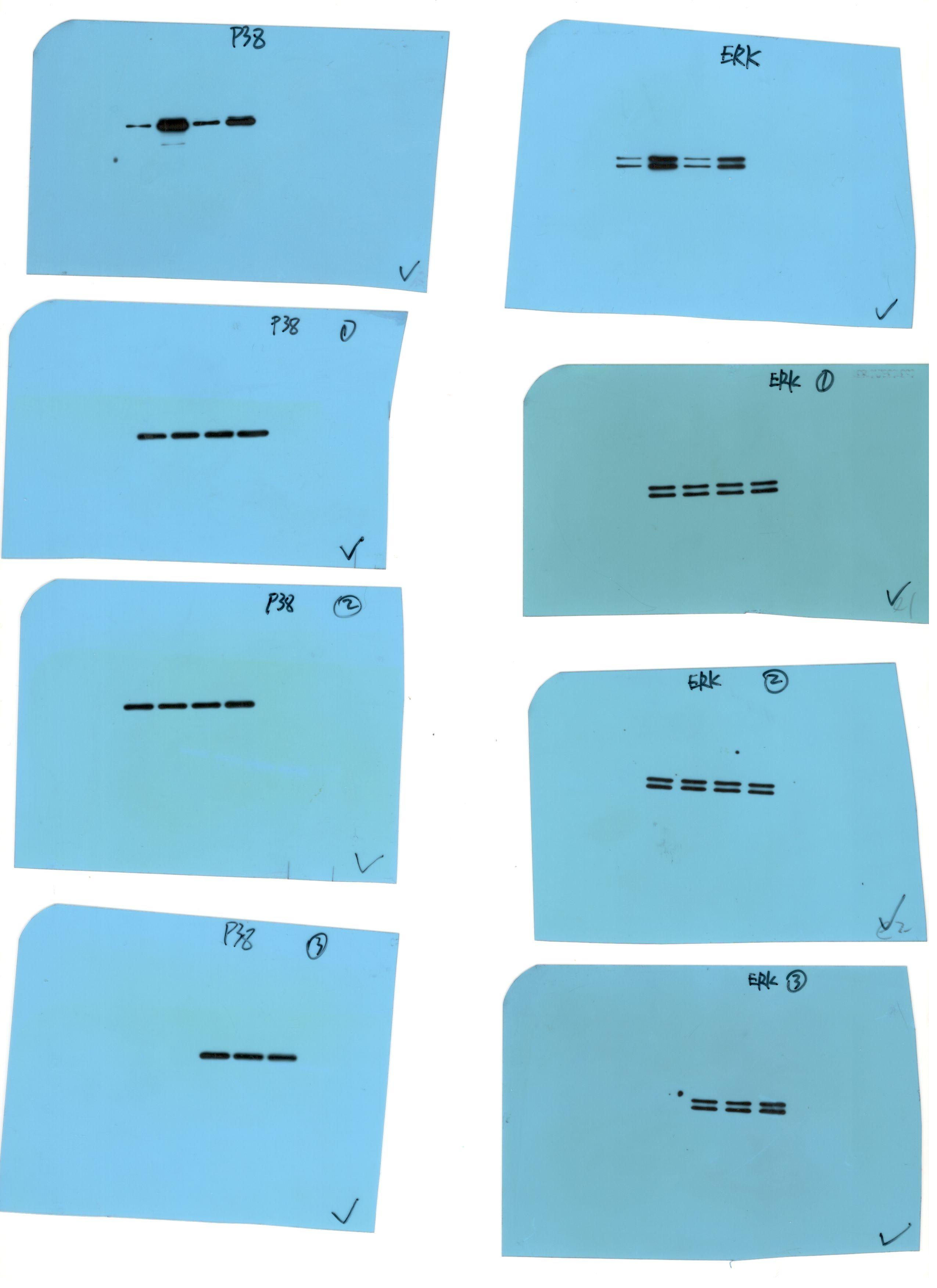

Supplement: Supplementary file 14 [file DataSheet2.ZIP › W-B/Figure 5 western-p-38 and ERK.jpeg]

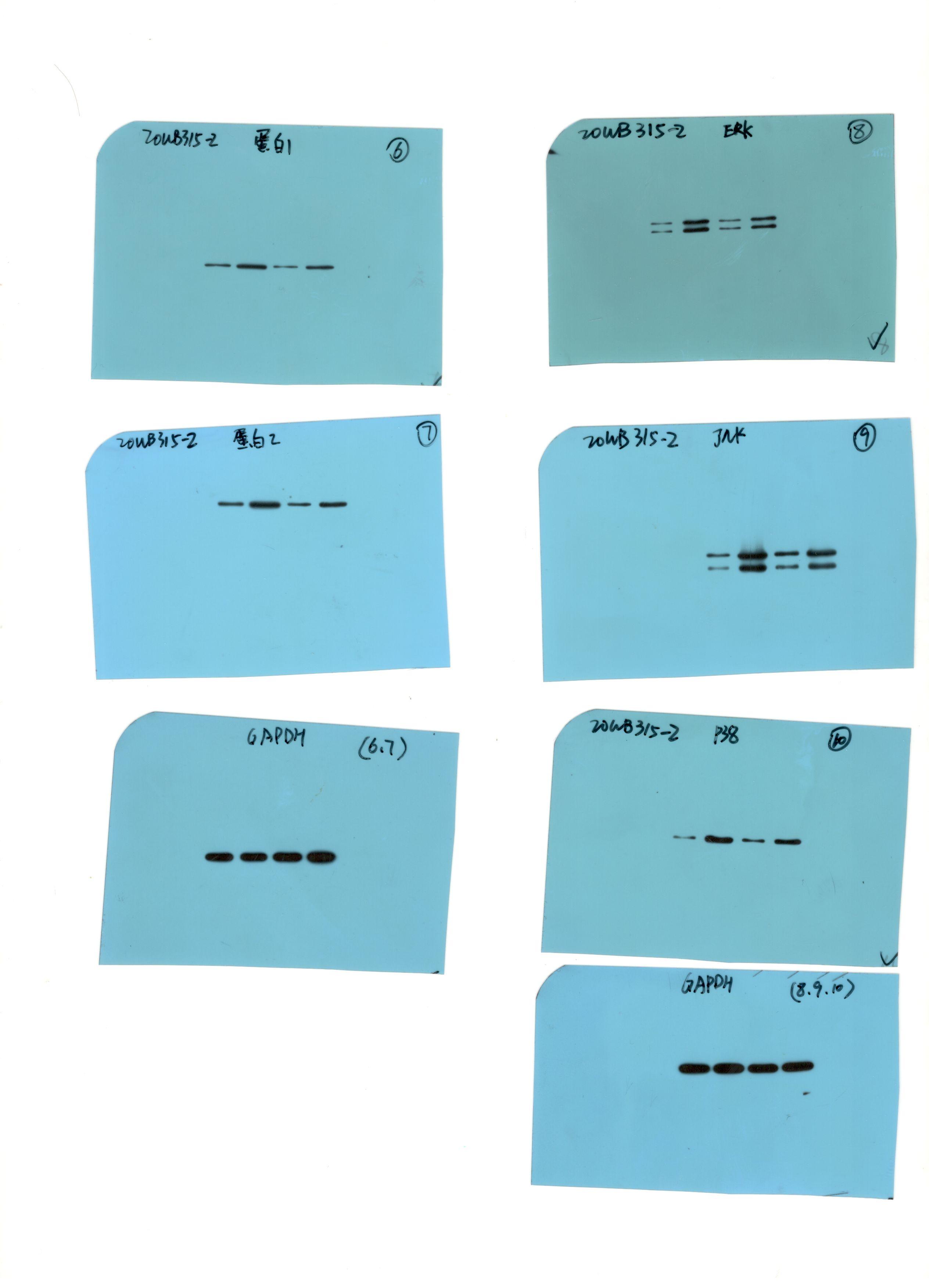

Supplement: Supplementary file 14 [file DataSheet2.ZIP › W-B/Figure 3 western blot .jpeg]

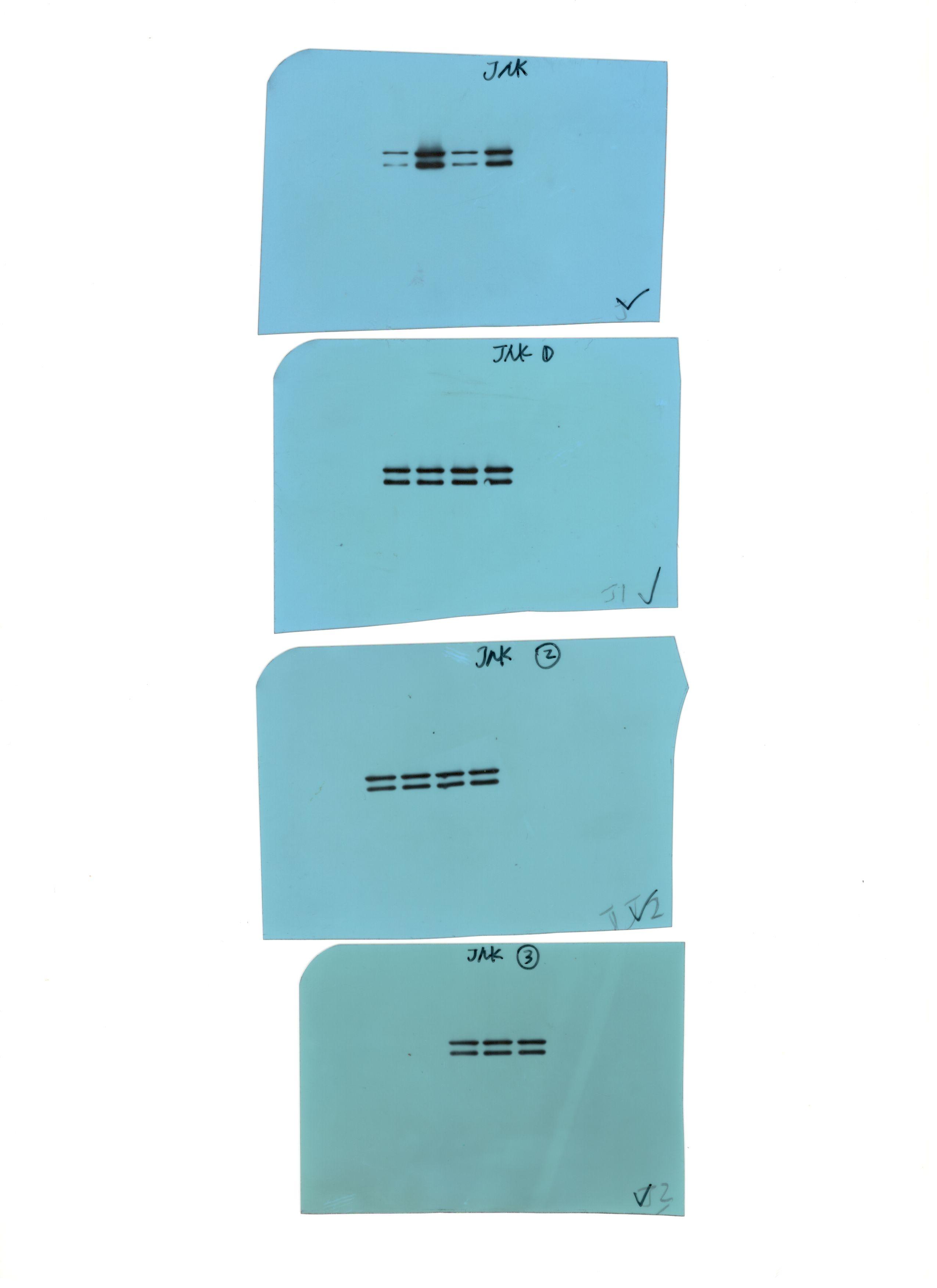

Supplement: Supplementary file 14 [file DataSheet2.ZIP › W-B/Figure 5 western JNK.jpeg]

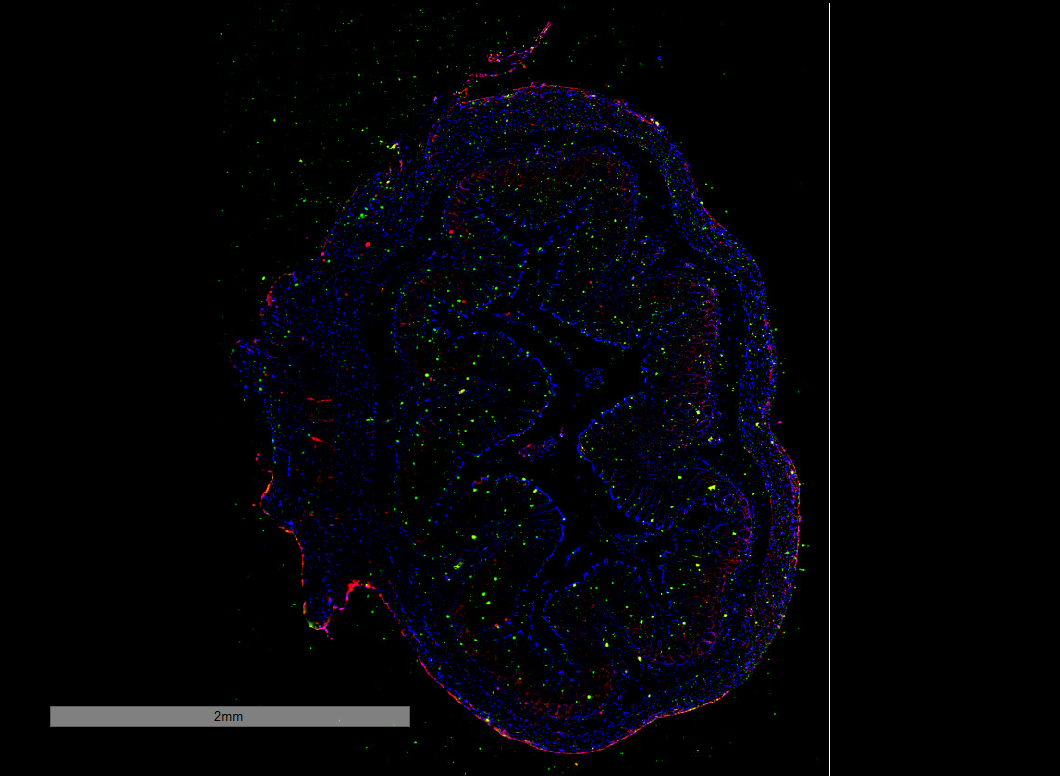

Supplement: Supplementary file 15 [file Image8.TIF]

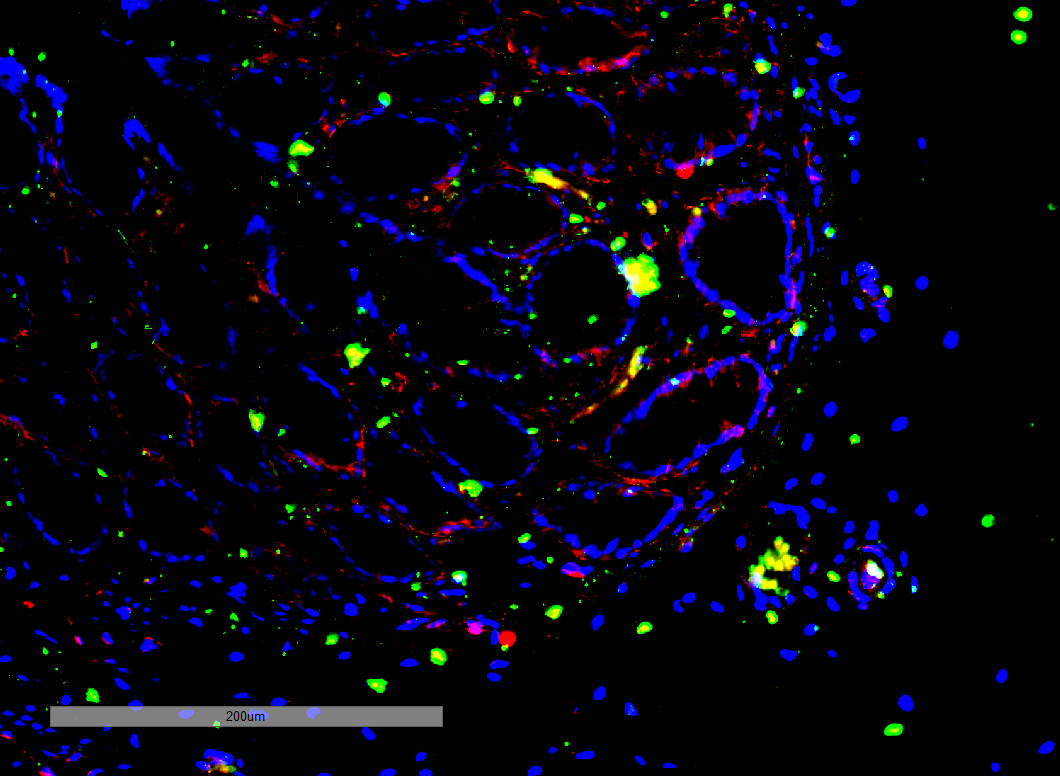

Supplement: Supplementary file 16 [file Image5.TIF]

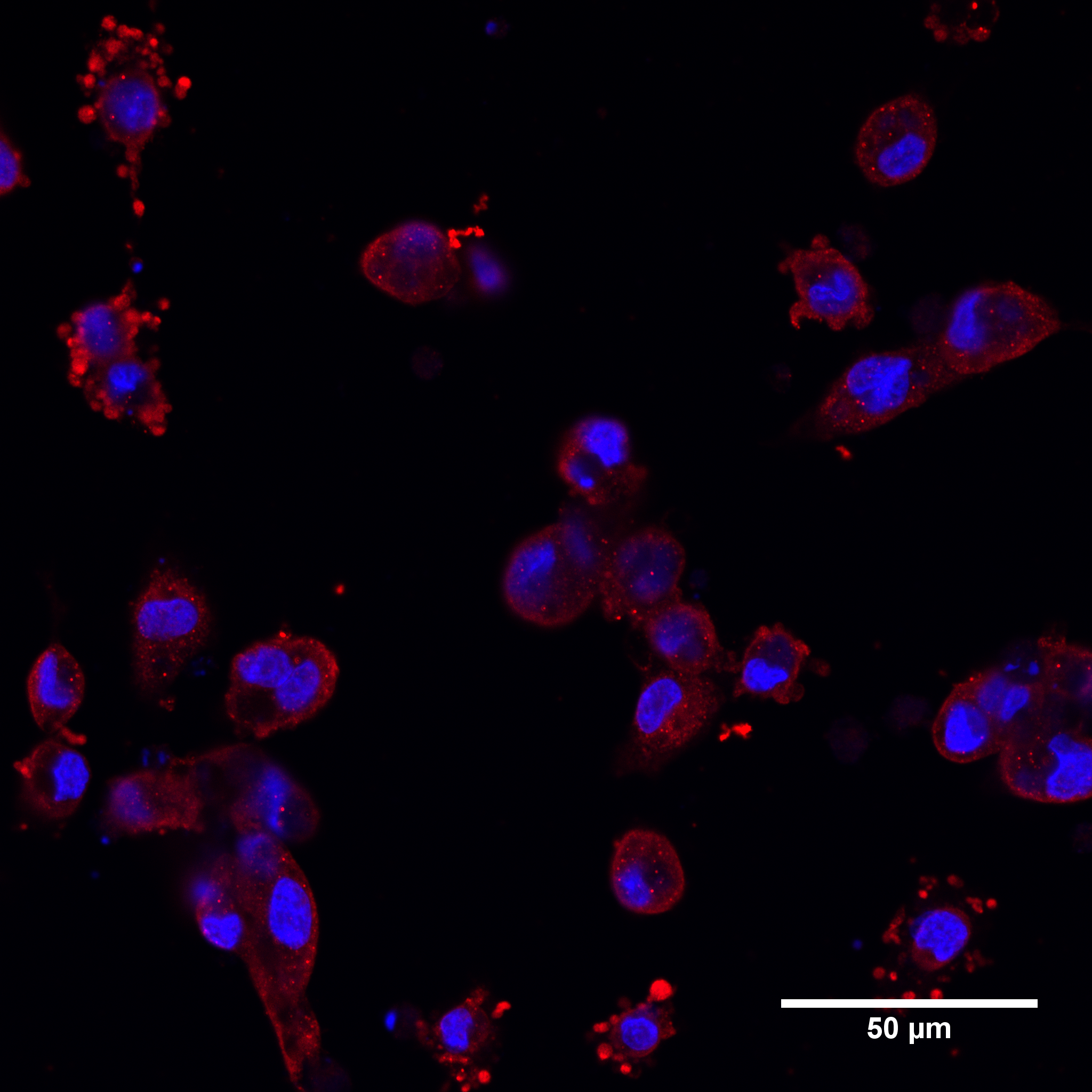

Supplement: Supplementary file 17 [file Image12.TIF]
